# Effects of the COVID-19 pandemic on primary care-recorded mental illness and self-harm episodes in the UK: a population-based cohort study

**DOI:** 10.1016/S2468-2667(20)30288-7

**Published:** 2021-01-11

**Authors:** Matthew J Carr, Sarah Steeg, Roger T Webb, Nav Kapur, Carolyn A Chew-Graham, Kathryn M Abel, Holly Hope, Matthias Pierce, Darren M Ashcroft

**Affiliations:** a Centre for Pharmacoepidemiology and Drug Safety, University of Manchester, Manchester, UK; b National Institute for Health Research Greater Manchester Patient Safety Translational Research Centre, University of Manchester, Manchester, UK; c Centre for Mental Health and Safety, University of Manchester, Manchester, UK; d Centre for Women's Mental Health, University of Manchester, Manchester, UK; e Manchester Academic Health Science Centre, Manchester, UK; f Greater Manchester Mental Health NHS Foundation Trust, Manchester, UK; g School of Medicine, Keele University, Keele, UK

## Abstract

**Background:**

The COVID-19 pandemic has adversely affected population mental health. We aimed to assess temporal trends in primary care-recorded common mental illness, episodes of self-harm, psychotropic medication prescribing, and general practitioner (GP) referrals to mental health services during the COVID-19 emergency in the UK.

**Methods:**

We did a population-based cohort study using primary care electronic health records from general practices registered on the UK Clinical Practice Research Datalink (CPRD). We included patient records from Jan 1, 2010, to Sept 10, 2020, to establish long-term trends and patterns of seasonality, but focused primarily on the period January, 2019–September, 2020. We extracted data on clinical codes entered into patient records to estimate the incidence of depression and anxiety disorders, self-harm, prescriptions for antidepressants and benzodiazepines, and GP referrals to mental health services, and assessed event rates of all psychotropic prescriptions and self-harm. We used mean-dispersion negative binomial regression models to predict expected monthly incidence and overall event rates, which were then compared with observed rates to assess the percentage reduction in incidence and event rates after March, 2020. We also stratified analyses by sex, age group, and practice-level Index of Multiple Deprivation quintiles.

**Findings:**

We identified 14 210 507 patients from 1697 UK general practices registered in the CPRD databases. In April, 2020, compared with expected rates, the incidence of primary care-recorded depression had reduced by 43·0% (95% CI 38·3–47·4), anxiety disorders by 47·8% (44·3–51·2), and first antidepressant prescribing by 36·4% (33·9–38·8) in English general practices. Reductions in first diagnoses of depression and anxiety disorders were largest for adults of working age (18–44 and 45–64 years) and for patients registered at practices in more deprived areas. The incidence of self-harm was 37·6% (34·8–40·3%) lower than expected in April, 2020, and the reduction was greatest for women and individuals aged younger than 45 years. By September, 2020, rates of incident depression, anxiety disorder, and self-harm were similar to expected levels. In Northern Ireland, Scotland, and Wales, rates of incident depression and anxiety disorder remained around a third lower than expected to September, 2020. In April, 2020, the rate of referral to mental health services was less than a quarter of the expected rate for the time of year (75·3% reduction [74·0–76·4]).

**Interpretation:**

Consequences of the considerable reductions in primary care-recorded mental illness and self-harm could include more patients subsequently presenting with greater severity of mental illness and increasing incidence of non-fatal self-harm and suicide. Addressing the effects of future lockdowns and longer-term impacts of economic instability on mental health should be prioritised.

**Funding:**

National Institute for Health Research and Medical Research Council.

## Introduction

The COVID-19 pandemic has had major implications for population mental health,^1^, ^2^, ^3^, ^4^ and it is estimated that up to 10 million people in England will require new or additional mental health support as a result of the pandemic.^5^ A national lockdown was implemented in the UK on March 23, 2020, with measures eased gradually from May, 2020. Although the first local lockdown was introduced in late June, 2020, social restrictions were reduced in most areas of the UK from June to August, 2020. In September, 2020, the number of confirmed COVID-19 cases began to increase and new restrictions to control transmission of the virus were implemented in October, 2020, in England, Northern Ireland, Scotland, and Wales. These measures to contain the virus have resulted in widespread societal disruption and economic downturn.^4^ Following previous economic recessions, the incidence and prevalence of mental illness, self-harm, and suicide has increased,^6^, ^7^ although the full economic impact of the COVID-19 pandemic is not yet known.

Evidence from self-report surveys indicates that short-term increases in the prevalence of mental illness, self-harm, and suicidal ideation might have occurred after implementation of the UK lockdown in March, 2020.^8^, ^9^, ^10^, ^11^ Such increases are concerning because non-fatal self-harm is a strong risk factor for suicide.^4^, ^12^ Direct comparison of prevalence of mental health disorders before and after the COVID-19 pandemic is challenging; however, available evidence indicates that, to date, the prevalence of anxiety and depression during the pandemic has been higher than would be expected for the general population.^9^ Public health messages in the UK initially encouraged patients to avoid attending general practices and hospitals to help control the virus. WHO reported substantial disruptions to mental health services in 130 countries.^13^ In Salford, England, compared with expected levels, presentations for common mental illnesses declined by 50% between March and May, 2020.^14^ Understanding how primary care-recorded mental illness and self-harm was affected by the various stages of the COVID-19 pandemic will indicate the extent of potential unmet need among specific patient groups.

**Research in context**

**Evidence before this study**

We searched Web of Science and PubMed from database inception to Oct 16, 2020, for articles published in English using the search terms “TITLE (covid* OR coronavirus OR sars-cov-2) AND TITLE or ABSTRACT (suic* OR self-harm* OR self-injur* OR self-poison* OR depress* OR anxi* OR mental* OR psych*)”. We also searched the medRxiv server for preprints of articles in the primary care research and psychiatry and clinical psychology sections. We did additional searches of the websites of public sector bodies for relevant reports. International evidence from surveys suggests that depression, anxiety disorders, and self-harm have become more common since the start of the COVID-19 pandemic. However, surveys of clinicians and analysis of local electronic health records have found substantial reductions in clinical presentations of mental illness. To date, no evidence is available on national presentation rates for specific mental health disorders among demographic subgroups of patients.

**Added value of this study**

To our knowledge, this is the first population-based study to assess the effect of COVID-19 on primary care-recorded mental illness and self-harm in the UK. On the basis of data from more than 14 million patients registered in general practices across the four nations of the UK, we found substantial reductions in first diagnoses of depression and anxiety disorders and incident self-harm compared with expected rates. Reductions were largest for adults of working age, women and younger people (aged <45 years) seeking help for self-harm, and patients registered at practices in areas with higher levels of deprivation. By September, 2020, the incidence of primary care-recorded self-harm and mental illness had returned to, or was close to, expected rates in England.

**Implications of all the available evidence**

Despite evidence of increased mental health burden due to the COVID-19 pandemic, marked reductions in primary care contact for mental illness were observed from April, 2020. Consequences of this unmet need could include increases in severity of mental illness, increases in self-harm and suicide rates, and widening of existing health inequalities. Addressing delays in diagnosis and management could help avoid increased burden of mental illness, particularly in adults of working age, among women and younger people seeking help for self-harm, and patients living in deprived areas. Health services need to be aware of potential reductions in patient contact as the UK enters subsequent lockdowns and of possible increases in demand for mental health care in the future.

Identifying gaps in mental health care has been specified as an urgent research priority in the response to COVID-19.^1^ There are specific concerns for certain demographic groups, including those at elevated risk of severe illness from COVID-19, such as older adults and people with underlying predisposing health conditions.^1^ Usual clinical care and social support for people with pre-existing mental illnesses are likely to have been disrupted, which could exacerbate symptoms.^1^ Young adults are also at risk from deterioration in their mental health,^9^, ^11^, ^15^ although some studies have reported improved mental health during lockdown among this age group.^16^ Increased unemployment and financial insecurity are likely to have more severe ramifications for socially disadvantaged groups than less disadvantaged groups,^2^ leading to concerns that the pandemic could widen pre-existing inequalities. In the UK, rates of COVID-19 infection have been disproportionately higher in deprived communities.^15^, ^17^ To date, most evidence regarding the mental health impact of COVID-19 at the national level is from self-report surveys.^11^, ^18^ However, individuals with pre-existing mental illnesses and older adults are less likely to complete such surveys, which could potentially mask the effects of COVID-19 on these groups.^18^

In this study, we used a large primary care longitudinal dataset, broadly representative of the UK population, to investigate the incidence of primary care-recorded common mental illnesses, self-harm, psychotropic medication prescribing, and general practitioner (GP) referrals to mental health services, and event rates of all psychotropic medication prescribing and self-harm episodes between January, 2019, and September, 2020. We also assessed the provision of primary care for mental illness across different population subgroups during the emergency.

## Methods

### Study design, data sources, and participants

For this population-based cohort study, we used primary care electronic health records obtained from the Clinical Practice Research Datalink (CPRD) Aurum and GOLD databases.^19^, ^20^ We included patient records from Jan 1, 2010, to Sept 10, 2020. Pre-pandemic data were included to establish long-term trends and patterns of seasonality; however, we focused primarily on reporting observed versus expected rates between January, 2019, and September, 2020. CPRD Aurum includes primary care data from contributing general practices in England that use the EMIS clinical system, and CPRD GOLD includes data from contributing practices in all four UK nations and is extracted from the Vision system. The CPRD GOLD dataset is broadly representative of the UK population with regards to age and sex,^19^ and CPRD Aurum is broadly representative of geographical coverage, area-level deprivation, age, and sex in England.^20^ The CPRD contains anonymised consultation records and includes patient demographic information, symptoms, diagnoses, medication prescriptions, and referrals to secondary care. We also obtained practice-level data on Index of Multiple Deprivation (IMD) quintiles (2014 IMD for Wales; 2015 IMD for England; 2016 IMD for Scotland; 2017 IMD for Northern Ireland).^21^ The IMD indicates relative deprivation at the area level, and is derived from several domains and aggregated as a single composite score that is ranked within each UK nation.

All individuals aged 10 years and older were eligible for inclusion. For each patient, we defined a so-called period of eligibility for study inclusion, which commenced on the latest of: the study start date (Jan 1, 2010); the patient's most recent registration with their practice; or the date on which data from the practice was deemed up-to-standard by the CPRD.^19^ A patient's period of eligibility ended on the earliest of: registration termination; the end of data collection from their practice; or death. For incident cases, we also applied a retrospective analysis period during which a patient was required to have been registered for at least 1 year before an incident episode.

The study was approved by the Independent Scientific Advisory Committee for CPRD research (20_094R1). All patient data were de-identified; thus, the requirement for patient consent was waived. Individual patients can opt out of sharing their records with the CPRD. A summary of the study protocol is available online.

### Outcomes

The outcomes of the study were incidence of specific common mental illness diagnoses (depression and anxiety disorders), prescriptions for antidepressants and benzodiazepines (the most commonly prescribed psychotropic medication types), GP referrals to mental health services (such as clinical psychology, psychotherapy, and outpatient mental health services), and episodes of self-harm. Information on referrals to mental health services was available only for practices in Northern Ireland, Scotland, and Wales (obtained from the GOLD dataset). Further outcomes were event rates of all psychotropic prescriptions and self-harm. We did not assess event rates for depression or anxiety disorders because typically GPs only code longer-term conditions once. Therefore, patients might subsequently visit with symptoms of depression or anxiety, but without additional diagnostic coding.

We used a broad definition of self-harm that captured episodes of varying suicidal intent, which aligned with the definition used in UK National Institute for Health and Care Excellence guidance.^22^ Considering the relatively large number of hospital presentations for self-harm in the UK, this outcome is likely to include higher proportions of secondary care-treated episodes in our study. We included all primary care-recorded codes for depression, anxiety, and self-harm, some of which would have been added to a patient's record following a hospital presentation or outpatient appointment. Mental illness, self-harm episodes, and prescriptions for psychotropic medication were identified from Read/SNOMED/EMIS codes used in CPRD GOLD and CPRD Aurum databases. All code lists were verified by two senior clinical academics (CAC-G, NK) and medication lists were reviewed by a senior academic pharmacist (DMA).

The first recorded code for each outcome category (anxiety, depression, self-harm, antidepressant or benzodiazepine prescription, and mental health referral) was included as an incident episode. Event rates included all specified codes for multiple self-harm or antidepressant or benzodiazepine prescriptions recorded in patients' primary care records, regardless of whether the code was the individual's first or subsequent recorded code of that type. Individuals could contribute to the incidence counts for more than one outcome (eg, mental illness diagnosis, psychotropic medication prescription, or self-harm episode). The denominator for both incidence and event rates was the aggregate person-months at risk for the whole population or subgroup of interest. The denominator for estimating rates of first referral to mental health services was person-months among patients diagnosed with depression or an anxiety disorder or a recorded episode of self-harm on or before referral date. Incidence and overall event rates were stratified by sex, age group (10–17, 18–44, 45–64, 65–79, and ≥80 years), and practice-level deprivation (IMD quintiles).

### Data analysis

The Aurum and GOLD databases were analysed separately, with data from Aurum restricted to English practices and GOLD providing information on practices in Northern Ireland, Scotland, and Wales. We structured data in a time-series format with incident and all event counts and person-months at risk aggregated (by year and month) with stratification by sex, age group, and deprivation quintile.

We used mean-dispersion negative binomial regression models to estimate expected monthly incident and all event counts from March, 2020, on the basis of predicted rates using data collected in the 10 years before the pandemic (Jan 1, 2010–Feb 29, 2020). The natural logarithm of the denominator (person-months at risk) was used as an offset in each regression model. To account for possible seasonality and long-term linear trends, we fitted calendar month as a categorical variable and time as a continuous variable with the number of months since the start of the study as the unit of measurement. For each month studied, observed and expected incident and all event counts were converted to rates using the observed person-month denominator. We plotted monthly expected rates and corresponding 95% CIs against the observed rates. Since the rates shared a common denominator, differences between expected and observed monthly rates were expressed as the relative rate reduction, and were calculated by subtracting the observed incident and all event counts from the predicted counts and dividing by the predicted counts. Relative rate reductions in incident and event rates were calculated for two time periods: March 1–Sept 10, 2020 (to represent the time from when the pandemic began to have an effect on primary care-recorded mental illness and self-harm to the end of our study period), and April 1–May 1, 2020 (to represent the first month after the initial reduction in primary care-recorded mental illness and self-harm was observed). Relative rate reductions are expressed as percentages with 95% CIs. The cohort was restricted to patients with records that were deemed acceptable by the CPRD for research purposes, which excluded patients with missing data on sex or age. IMD data were missing for 7·8% of the study population in CPRD Aurum and 4·5% of the study population in CPRD GOLD (appendix p 15). We excluded practices with missing IMD data from IMD-stratified analyses; to check for effects of missing data, we did a post-hoc analysis of incidence for patients from practices with versus those without missing IMD data. Data analysis was done using Stata (version 16).

This study was done in accordance with REporting of studies Conducted using Observational Routinely-collected health Data guidance (appendix p 20).^23^

A panel of service users and carers with lived experience of mental health services worked with the research team to interpret the study's results. The group is linked with the National Institute for Health Research Greater Manchester Patient Safety Translational Research Centre. We used GRIPP2-SF reporting checklists to report service user involvement (appendix p 16).^24^

### Role of the funding source

The study funders had no role in the study design, data collection, data analysis, data interpretation, or writing of the report. All authors had full access to the data reported in the study and the final responsibility to submit for publication.

## Results

The study population included 11 946 696 patients from 1362 general practices in England and 2 263 811 patients from 335 general practices in Northern Ireland, Scotland, and Wales. 84 general practices in England included in the GOLD database were excluded from the study to avoid overlap between any practices that switched system to prevent duplication of data from practices that might be included in both databases. 24 897 725 patients (21 308 886 from CPRD Aurum; 3 588 839 from CPRD GOLD) contributed data for the estimation of the expected rates in the pre-COVID-19 comparison period (Jan 1, 2010–Feb 29, 2020). Detailed demographic and summary data, including person-time (used to model trends in expected rates and to compare observed and expected rates) and total numbers of diagnoses and events analysed for the study cohorts, are included in the appendix (pp 17–18).

**Figure 1:**
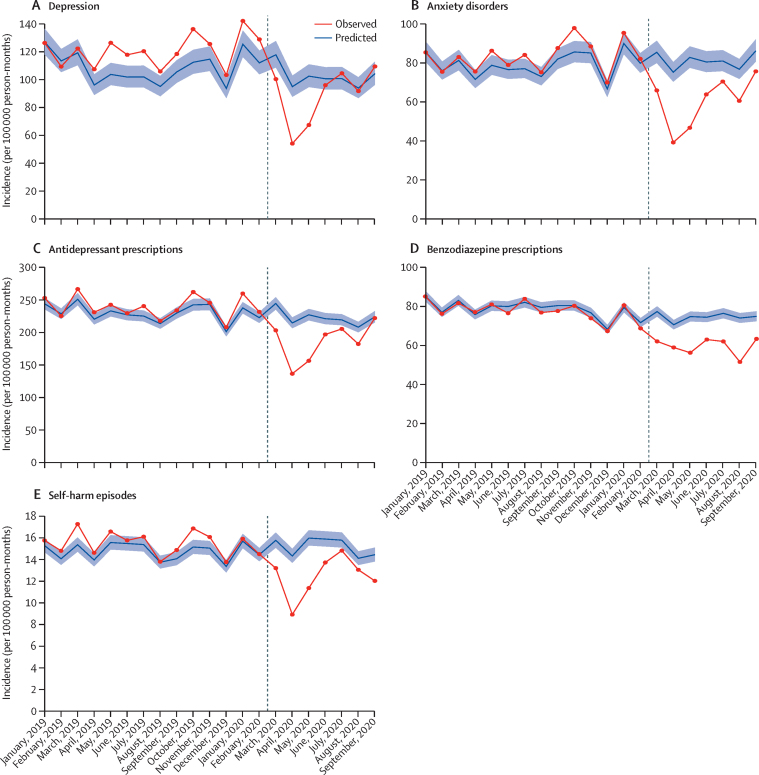
Expected and observed incidence of mental illness diagnoses, psychotropic medication prescriptions, and self-harm episodes in England (January, 2019–September, 2020) Vertical lines shows March 1, 2020. Shaded areas represent 95% CI.

In English general practices, in April, 2020, incident diagnoses of depression and anxiety disorders, antidepressant prescribing, and self-harm all decreased substantially compared with expected rates. In England, between April 1 and May 1, 2020, the incidence of primary care-recorded depression decreased by 43·0% (95% CI 38·3–47·4), anxiety disorders by 47·8% (CI 44·3–51·2), first antidepressant prescribing by 36·4% (33·9–38·8), and self-harm by 37·6% (34·8–40·3; table). Subsequently, incidence for diagnosis of depression and anxiety disorders, antidepressant prescribing, and self-harm increased in May and June, 2020, and by September, 2020, rates were similar to expected rates (figure 1). The decrease in incidence of benzodiazepine prescribing was less pronounced in April, 2020, and the incidence remained lower than expected up to September, 2020.

**Table:** Percentage reductions in incidence and event rates of common mental illness, self-harm, antidepressant and benzodiazepine prescribing, and referrals to mental health services in the UK between March 1 and Sept 10, 2020

		**March 1–Sept 10, 2020**			**April 1–May 1, 2020**		
		Observed frequency, n	Expected frequency, n	Percentage reduction (95% CI)	Observed frequency, n	Expected frequency, n	Percentage reduction (95% CI)
**England**							
Incidence (per 100 000 person-months)							
	Depression	53 443	62 032	13·8% (6·7 to 20·4)	4964	8724	43·0% (38·3 to 47·4)
	Anxiety disorders	38 189	51 947	26·4% (21·4 to 31·2)	3807	7305	47·8% (44·3 to 51·2)
	Antidepressants	93 845	113 575	17·3% (14·1 to 20·5)	10 512	16 537	36·4% (33·9 to 38·8)
	Benzodiazepines	36 762	46 239	20·4% (17·7 to 23·1)	5510	6602	16·5% (13·6 to 19·3)
	Self-harm	8512	10 388	18·0% (14·3 to 21·5)	920	1476	37·6% (34·8 to 40·3)
Event rate (per 100 000 person-months)							
	Antidepressants	7 237 716	7 573 286	4·4% (1·4 to 7·3)	1 031 928	1 120 229	7·8% (4·9 to 10·7)
	Benzodiazepines	818 541	853 967	4·1% (1·5 to 6·7)	121 786	126 669	3·8% (1·2 to 6·4)
	Self-harm	24 109	29 716	18·8% (15·8 to 21·7)	2666	4206	36·6% (34·2 to 38·8)
**Northern Ireland, Scotland, and Wales**							
Incidence (per 100 000 person-months)							
	Depression	4601	8714	47·1% (44·1 to 50·1)	451	1305	65·4% (63·4 to 67·3)
	Anxiety disorders	3655	6178	40·8% (38·1 to 43·3)	401	944	57·5% (55·5 to 59·3)
	Antidepressants	18 081	24 817	27·1% (24·3 to 29·8)	2093	3824	45·2% (43·1 to 47·2)
	Benzodiazepines	9342	11 480	18·6% (15·6 to 21·4)	1400	1706	17·9% (14·8 to 20·8)
	Self-harm	1808	1988	9·0% (2·8 to 14·8)	230	305	24·5% (19·2 to 29·4)
	Referral to mental health services	1337	2540	47·3% (44·7 to 49·8)	97	393	75·3% (74·0 to 76·4)
Event rate (per 100 000 person-months)							
	Antidepressants	1 721 824	1 803 768	4·5% (1·7 to 7·2)	257 671	278 751	7·5% (4·9 to 10·1)
	Benzodiazepines	273 154	277 139	1·4% (−1·1 to 3·9)	42 717	42 996	0·6% (−1·9 to 3·2)
	Self-harm	4433	4747	6·6% (1·2 to 11·7)	526	716	26·5% (22·3 to 30·6)

When considering event rates for all prescribing and self-harm episodes in English practices (ie, not just patients' first consultation of this type), the majority were for patients with a history of mental illness or self-harm. For example, 10 512 (1%) of 1 031 928 antidepressant prescriptions in April, 2020, were first prescriptions. For the period April 1 to May 1, 2020, the event rates for antidepressant and benzodiazepine prescribing were slightly lower than expected (7·8% relative rate reduction [95% CI 4·9–10·7 for antidepressants; 3·8% [1·2–6·4] for benzodiazepines; table; appendix p 2). Although the event rates for self-harm increased in May and June, 2020, rates remained lower than expected levels in September, 2020 (appendix p 2).

Temporal changes in the incidence of primary care-recorded depression, anxiety disorders, self-harm and prescribing in Northern Ireland, Scotland, and Wales differed from those in England. The incidence of depression and anxiety disorders increased after April, 2020, but rates remained around a third lower than expected until September, 2020 (appendix p 6). Furthermore, the cumulative difference between observed and expected rates of incident primary care-recorded depression and anxiety and first antidepressant prescribing between April and September, 2020, was larger in practices from Northern Ireland, Scotland, and Wales than England (table). Although the incidence of self-harm increased to expected rates in June and July, 2020, a reduction was observed in August and September, 2020 (appendix p 6). In April, 2020, the observed rate of referral to mental health services was less than a quarter of the expected rate for the time of year (75·3% reduction [95% CI 74·0–76·4; table; appendix p 6). Referral rates increased between May and August, 2020, but remained considerably lower than the expected rates for September, 2020.

**Figure 2:**
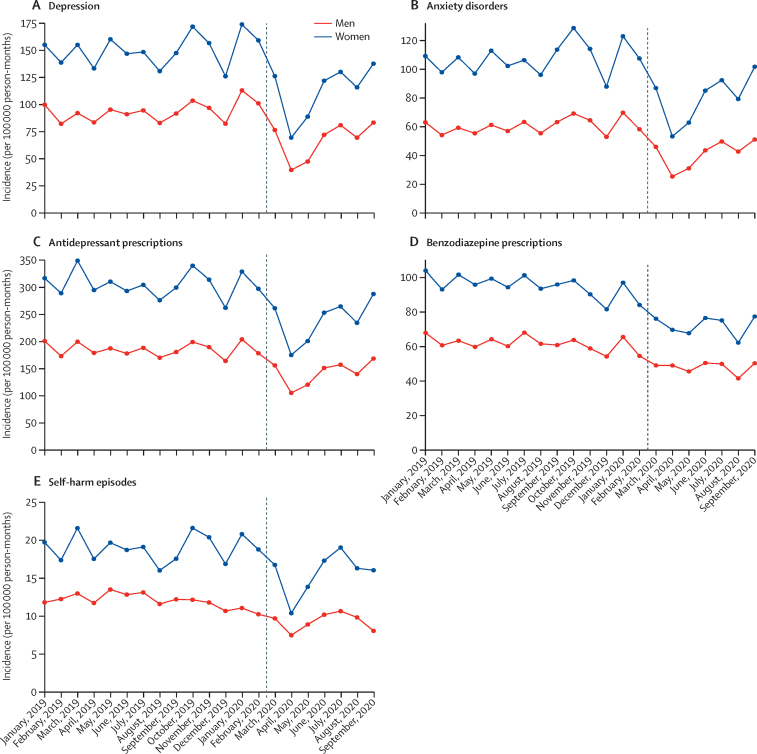
Incidence of mental illness diagnoses, psychotropic medication prescriptions, and self-harm episodes in England, stratified by sex (January, 2019–September, 2020) Vertical lines shows March 1, 2020.

**Figure 3:**
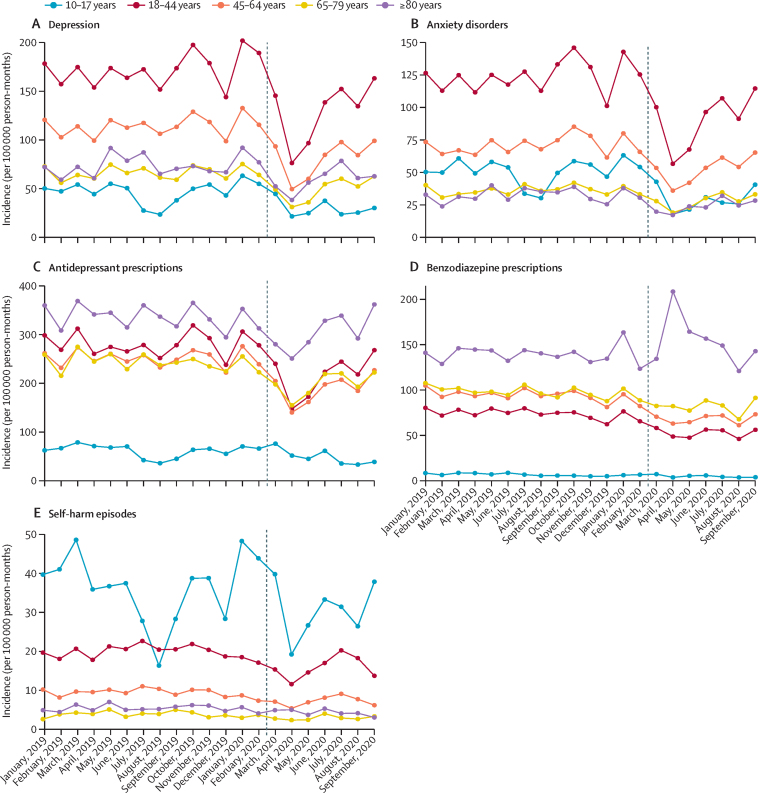
Incidence of mental illness diagnoses, psychotropic medication prescriptions, and self-harm episodes in England, stratified by age group (January, 2019–September, 2020) Vertical lines show March 1, 2020.

**Figure 4:**
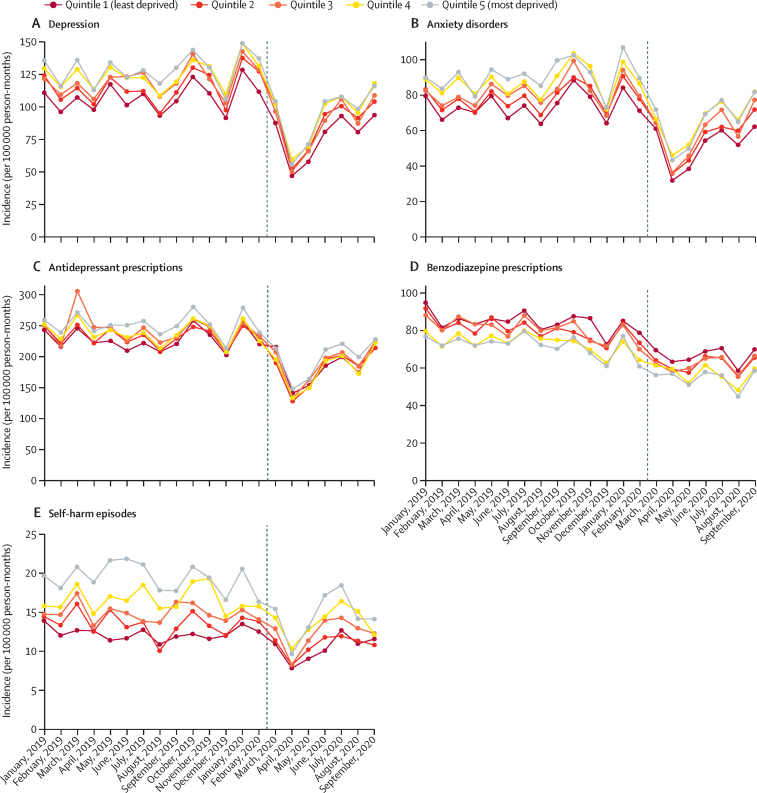
Incidence of mental illness diagnoses, psychotropic medication prescriptions, and self-harm episodes in England, stratified by Index of Multiple Deprivation quintiles (January, 2019–September, 2020) Vertical lines show March 1, 2020.

In England, patterns of incidence and overall event rates for depression, anxiety disorders, and psychotropic medication prescribing were similar for men and women, although rates were consistently higher among women than men for all outcomes (figure 2; appendix p 3). In April, 2020, observed incidence and event rates for self-harm were substantially lower than expected for women and people aged younger than 45 years (figure 3). Self-harm incidence increased from August, 2020, in the 10–17-year age group (figure 3). Adults of working age (18–44 and 45–64 years) had the largest absolute decreases and relative reductions in primary care-recorded diagnosis of depression and anxiety disorders in April, 2020 (figure 3). A marked increase was observed in first benzodiazepine prescribing for adults aged 80 years and older in April 2020, after which rates gradually returned to expected levels (figure 3). The incidence of depression, anxiety disorders, and self-harm decreased for all practice-level IMD quintiles from April, 2020, with the largest reductions observed in the most deprived populations (figure 4). In a post-hoc analysis, practices with missing IMD data had similar rates of mental illness, self-harm, and prescribing to practices where IMD was known (appendix p 14). By September, 2020, the incidence of depression and anxiety disorders in all deprivation quintiles had increased. In Northern Ireland, Scotland, and Wales, practices in less deprived areas had the highest rates of referral to mental health services before March, 2020, and the largest subsequent reductions from April, 2020 (data not available for English practices; appendix p 9).

## Discussion

Following the onset of the COVID-19 pandemic, in April, 2020, the incidence of primary care-coded depression, anxiety disorders, antidepressant prescribing, and self-harm decreased sharply compared with expected rates in all four UK nations. Rates in England increased in May and June, 2020, and by September, 2020, were similar to expected rates. In Northern Ireland, Scotland, and Wales, a larger reduction in rates persisted until September, 2020. Initial decreases in incidence of self-harm were higher in women and individuals younger than 45 years, and adults of working age had the largest reductions in primary care-recorded depression and anxiety disorders. Decreases in the incidence of depression, anxiety disorders, and self-harm were observed for all practice-level IMD quintiles from April, 2020, with the largest reductions observed in the most deprived populations.

In early March, 2020, health services were required to balance infection control with access to care for patients, and GPs were advised to minimise the number of face-to-face patient contacts.^25^ The initial recovery in rates of primary care contact after May, 2020, among all demographic groups, towards expected rates by September, 2020, suggests that GPs adapted rapidly to increasing demands for care. We found that the event rates for antidepressant and benzodiazepine prescribing decreased slightly in the months after the COVID-19 pandemic began. Electronic repeat prescribing is likely to have mitigated against larger decreases in prescriptions. This finding could also indicate that some patients might have contacted their GP to discuss medication needs in preparation for the lockdown. The increase in benzodiazepine prescribing in patients aged 80 years and older in April, 2020, might reflect increased anxiety associated with fear of COVID-19 infection, changes to care home environments, and the effect of shielding in this age group. We observed reductions in help seeking for adults of working age, self-harm among women and younger people, and those registered with practices located in more deprived areas—groups that have also been identified as experiencing greater deterioration in mental health due to the pandemic.^9^, ^10^, ^15^, ^26^

The COVID-19 pandemic has affected deprived communities in the UK disproportionately: COVID-19 mortality rates in the most deprived areas are twice as high as mortality rates in the least deprived areas.^27^ The restrictions on non-urgent care and the decreases in emergency department attendance are also likely to have affected socioeconomically disadvantaged groups to a greater extent.^26^ Our findings suggest that people from deprived communities were affected disproportionately: patients registered with practices in deprived areas were observed to have the largest decreases in primary care-recorded mental illness and self-harm and referrals to mental health services. By contrast, before March, 2020, people registered with practices located in more deprived areas had the highest incidence of depression, anxiety disorders, and self-harm. Using survey data, one study found that socioeconomic deprivation was associated with higher risks of depression and anxiety disorders in adults and young people during the COVID-19 pandemic compared with pre-pandemic levels.^11^ The inverse care law, which describes the combination of greater health care need among people living in deprived areas and the lack of additional availability of health services in these areas to meet increased demand,^28^ has previously been demonstrated in relation to primary care management of self-harm, with lower referral rates associated with higher incidence and higher levels of deprivation.^29^ Our findings suggest that COVID-19 has widened such inequalities, and have implications for general practices in deprived areas managing potential demand.

Our findings regarding event rates suggest a temporary but marked reduction in the number of people with existing mental illness accessing primary care following an episode of self-harm. Primary care is a vital resource and support for people with mental illness. One study of people who died by suicide found that primary care was the most commonly used point of contact in the week before suicide.^12^ Ensuring that access to treatment is available for people with mental illness, who might require regular contact and support, and for whom physical distancing restrictions might exacerbate symptoms, is crucial.^1^ Evidence from the South London and Maudsley National Health Service (NHS) Trust in London, UK, showed that caseloads for community mental health teams remained relatively stable between March and May, 2020, whereas a marked reduction in caseload was observed for home treatment teams.^30^ Consistent with our findings, this suggests that some people experiencing a mental health crisis, who would have been expected to receive treatment, did not access primary care during the COVID-19 restrictions.

Monitoring patterns in primary care recording of mental illness diagnoses and self-harm episodes after September, 2020, will provide crucial information on equity of care for specific health conditions and between patient groups. Obtaining such data will also help to assess how ongoing restrictions intended to limit the spread of COVID-19, alongside the potential impact on population mental health, continue to affect help seeking. This evidence will enable public mental health interventions to be targeted to groups most in need, a key mechanism for reducing the mental health impact of COVID-19.^1^ Although event rates in this study mostly represented people with existing mental illness, future work should focus specifically on this group, and on people with a history of self-harm. Survivors of COVID-19 are more susceptible to mental illness, fatigue, and so-called long COVID,^31^ and patients who received critical care are at particular risk of post-traumatic stress disorder.^32^ Future work should investigate these longer-term outcomes among COVID-19 survivors. Further research is also needed on the mental health effects of COVID-19 containment restrictions on adults aged 80 and older.

Previous research suggests that symptoms of mental illness can increase if treatment needs are not met.^33^, ^34^ Possible consequences of this unmet need include increased numbers of admissions to psychiatric units and presentations to emergency departments for mental illness, self-harm, and drug and alcohol misuse, and heightened suicide risk. Ongoing monitoring to assess whether rates continue to increase beyond expected levels is important for ensuring health services can meet future demand. Further research using linkages to NHS Hospital Episode Statistics will enable examination of self-harm separately among people presenting to primary care versus those presenting to hospital emergency departments. It is unclear how community-based services and voluntary organisations, which are an important source of support for people with mental illness, were affected. Some organisations reported increased demand, whereas others had little change in service use.^35^ Additionally, the COVID-19 restrictions implemented in March, 2020, might have helped to reduce mental distress for some people, which might partly explain the reductions in contacts we observed. The potential for individuals to adapt to challenges and to experience post-traumatic growth in response to major upheaval also warrants further research.^36^ However, as the crisis persists into the winter months and beyond, there are likely to be greater challenges with regard to the management of economic and social impacts. Encouraging individuals to seek support from health services when needed, despite COVID-19 restrictions, is an important public health message. Patients might have accessed support elsewhere, such as from non-statutory services; the potential longer-term reductions in primary care contact for mental illness and self-harm, as populations adapt to changing provision of mental health support, is an important topic for future research.

The main strength of our study is the broadly nationally representative setting, which included more than 14 million patients registered with general practices across the four nations of the UK. The use of two discrete data sources, the Aarum and GOLD databases, also enabled independent replication in our study. Although there have been a number of self-report surveys evaluating changes in mental health since the COVID-19 crisis began, these are unlikely to capture effects on the most vulnerable groups such as those with existing mental illness.^18^ The surveys might also overestimate rates of mental illness and do not provide accurate information about clinical need.^18^

We acknowledge some limitations to our study. Recording of ethnicity within the CPRD was of insufficient quality to enable us to examine clinical contact among ethnic minority groups. Although we examined clinical contacts in all four nations of the UK, information on mental health referrals was not available in the CPRD data source for practices in England. Data from Northern Ireland, Scotland, and Wales are not fully representative of geographical coverage of patients and practice size; thus, findings might be less representative of smaller general practices.^19^ Patients from practices in the most deprived areas were over-represented in the study cohort, particularly in Northern Ireland and Wales. Although patterns observed for Northern Ireland, Scotland, and Wales were similar to those for England, the patient management software systems and the coding classification systems that contribute to the two CPRD databases used are different. Therefore, variation in the identification of mental illness and self-harm between the two databases in our study is possible. Some of the reduction in primary care-recorded mental illness and self-harm might have been a result of inaccuracies in coding due to the rapid changes and adaptations that GPs had to make, including a shift to remote consultation methods, during the early stages of the study period. Antidepressant and benzodiazepine medications have indications beyond treating mental illness, and therefore interpretation of findings associated with prescribing rates should take this into consideration. Although 98% of the UK population is registered at an NHS GP surgery, certain patients are not represented in our study, including prisoners, private patients, those in some residential homes, and some people with no fixed address.^19^, ^20^ Considering the relatively small incidence of self-harm compared with depression and anxiety disorders, and the potential delay in hospital-presenting self-harm episodes being added to patients' primary care records, it is possible that not all primary care-recorded self-harm would have been captured in the last two months of our study period (August and September, 2020).

The marked reduction in primary care-recorded mental illness and self-harm during the spring 2020 peak of the COVID-19 pandemic in the UK and the increased mental health burden resulting from the pandemic indicates untreated mental illness. Adults of working age, patients registered with practices located in deprived areas, and women and younger people who self-harm might have greater levels of undetected need. Addressing delays in diagnosis and treatment is a priority, particularly in these groups. Awareness of potential further reductions in help seeking and subsequent increases in the incidence of mental illness could help primary, secondary, and acute services prepare for changes in demand for mental health services.

## Data sharing

The clinical codes used in this study are available online. The codes are also available from the corresponding author on request. Access to data are available only once approval has been obtained through the individual constituent entities controlling access to the data. The primary care data can be requested via application to the Clinical Practice Research Datalink.
