# Supplementary material for: Effects of the COVID-19 pandemic on primary care-recorded mental illness and self-harm episodes in the UK: a population-based cohort study
Source: Lancet Public Health. 2021 Jan 11;6(2):e124–35. doi: 10.1016/S2468-2667(20)30288-7 (PMC7843955; doi:10.1016/S2468-2667(20)30288-7)
Supplement: Supplementary appendix [file mmc1.pdf]

THE LANCET
Public Health

Supplementary appendix

This appendix formed part of the original submission and has been peer reviewed. We post it as supplied by the authors.

Supplement to: Carr MJ, Steeg S, Webb RT, et al. Effects of the COVID-19 pandemic on primary care-recorded mental illness and self-harm episodes in the UK: a population-based cohort study. *Lancet Public Health* 2021; published online Jan 11. https://doi.org/10.1016/S2468-2667(20)30288-7.

Supplementary Material

Contents

Supplementary figures from CPRD Aurum representing general practices in England:

Page 2 **Figure S1:** Rates of psychotropic medication prescriptions and self-harm episodes

Page 3 **Figure S2:** Rates of psychotropic medication prescriptions and self-harm episodes – stratified by gender

Page 4 **Figure S3:** Rates of psychotropic medication prescriptions and self-harm episodes – stratified by age group

Page 5 **Figure S4:** Rates of psychotropic medication prescriptions and self-harm episodes – stratified by Index of Multiple Deprivation quintile

Supplementary figures from CPRD GOLD representing general practices in Northern Ireland, Scotland and Wales:

Page 6 **Figure S5:** Incident mental health diagnoses, psychotropic medication prescriptions and referrals to mental health services

Page 7 **Figure S6:** Incident mental illness diagnoses, psychotropic medication prescriptions and referrals to mental health services – stratified by gender

Page 8 **Figure S7:** Incident mental illness diagnoses, psychotropic medication prescriptions and referrals to mental health services – stratified by age group

Page 9 **Figure S8:** Incident mental illness diagnoses, psychotropic medication prescriptions and referrals to mental health services – stratified by Index of Multiple Deprivation quintile

Page 10 **Figure S9:** Rates of psychotropic medication prescriptions and self-harm episodes

Page 11 **Figure S10:** Rates of psychotropic medication prescriptions and self-harm episodes – stratified by gender

Page 12 **Figure S11:** Rates of psychotropic medication prescriptions and self-harm episodes – stratified by age group

Page 13 **Figure S12:** Rates of psychotropic medication prescriptions and self-harm episodes – stratified by Index of Multiple Deprivation quintile

Supplementary figure on Index of Multiple Deprivation

Page 14 **Figure S13:** Incident mental illness diagnoses, psychotropic medication prescriptions and self-harm episodes in England – stratified by an indicator for missing Index of Multiple Deprivation quintile

Supplementary tables

Page 15 **Table S1**: Index of Multiple Deprivation quintile of the study population by nation, on 1st March 2020

Page 16 **Table S2:** Involvement of people with lived experience: GRIPP2 Short-form checklist

Page 17 **Table S3:** Demographic characteristics for patients in the study population on 1st March 2020

Page 18 **Table S4a:** Person-time and total numbers of diagnoses/events/prescriptions analysed: practices in England

Page 18 **Table S4b:** Person-time and total numbers of diagnoses/events/prescriptions analysed: practices in Northern Ireland, Scotland and Wales

Page 19 **S5:** Supplementary information on the CPRD data source

Page 20 **Table S6**: RECORD Statement

Supplementary figures from CPRD Aurum representing general practices in England

Figure S1: Rates of psychotropic medication prescriptions and self-harm episodes (representing practices in England)

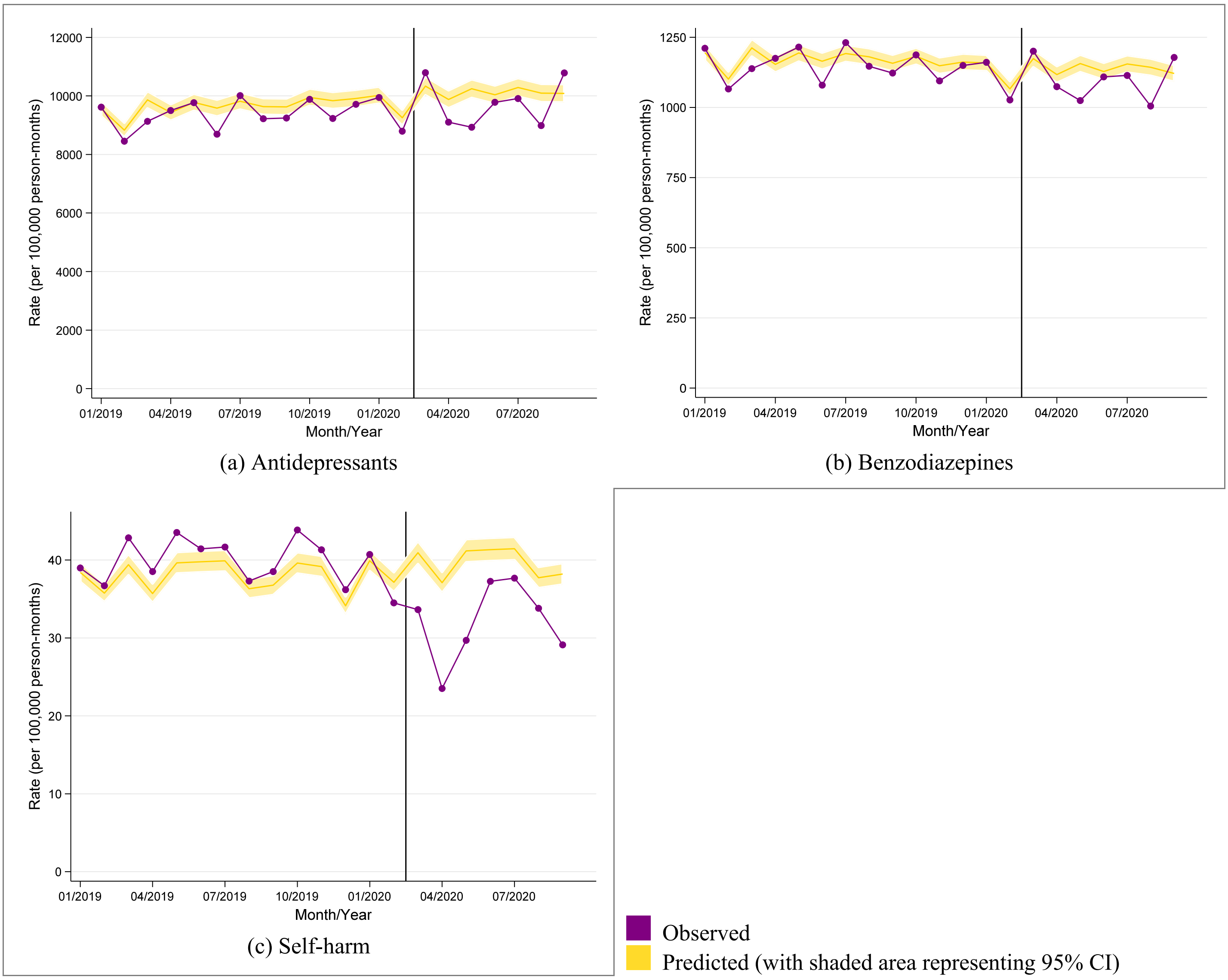

Figure S2: Rates of psychotropic medication prescriptions and self-harm episodes – stratified by gender (representing practices in England)

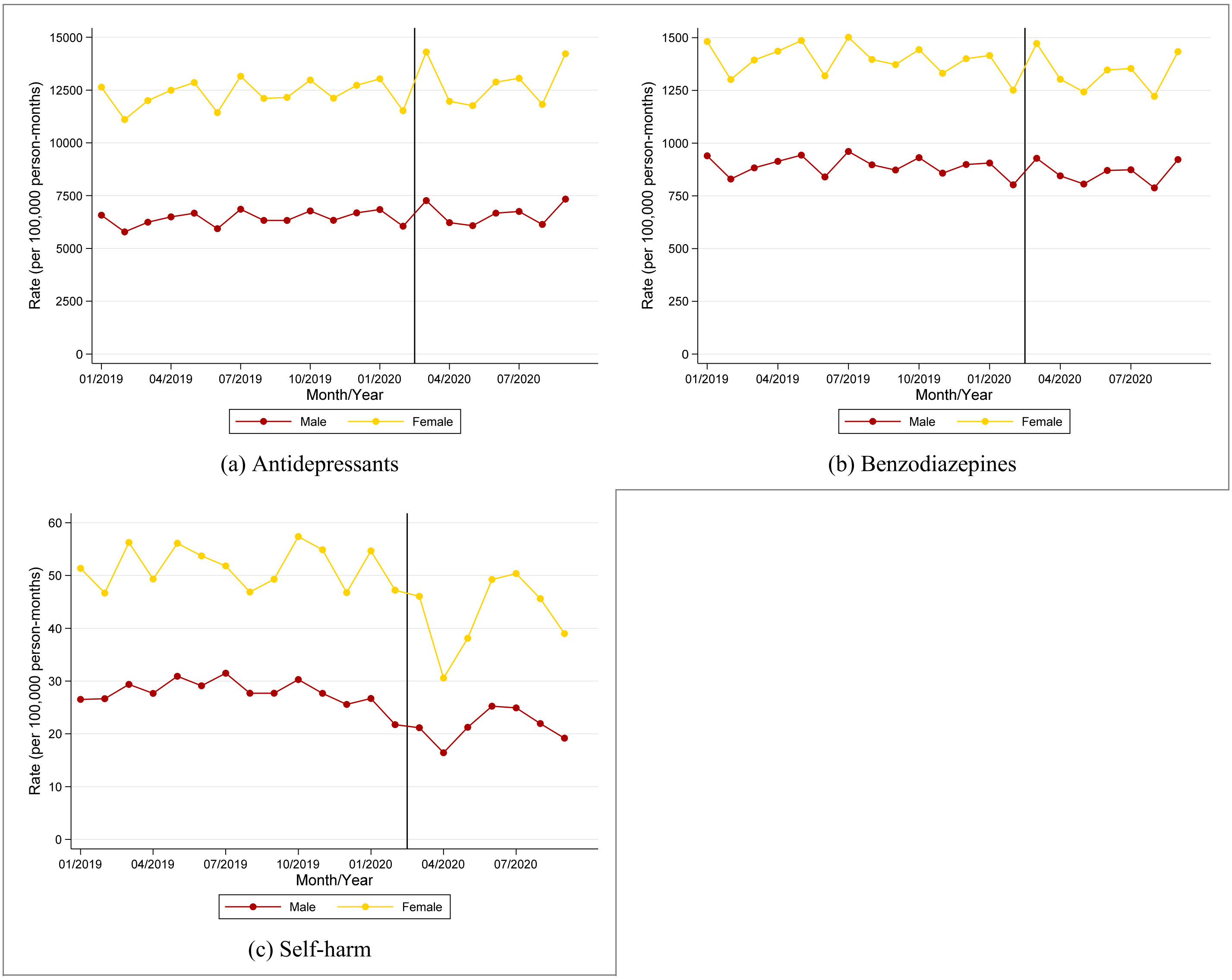

(a) Antidepressants

(b) Benzodiazepines

(c) Self-harm

Figure S3: Rates of psychotropic medication prescriptions and self-harm episodes – stratified by age group (representing practices in England)

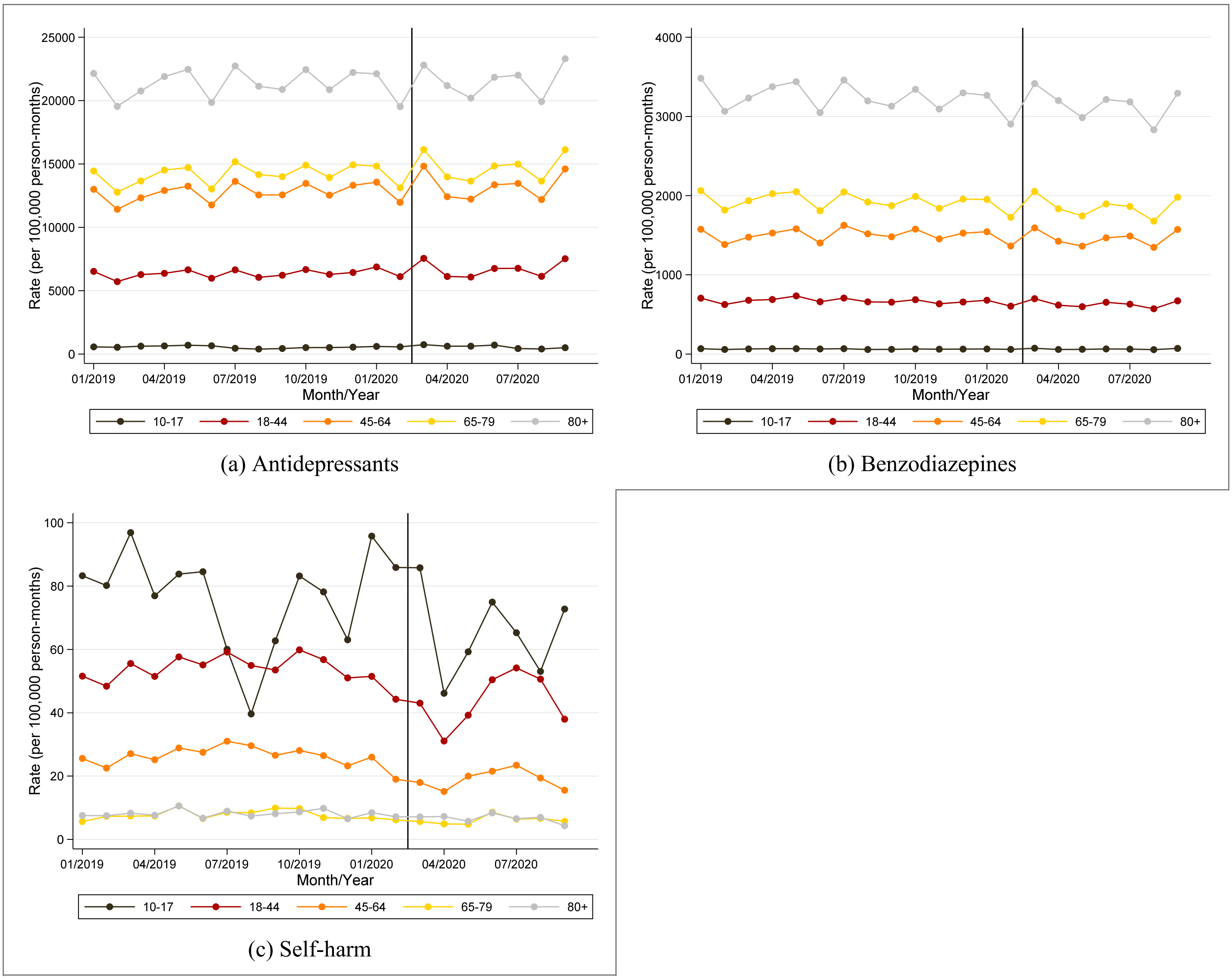

(a) Antidepressants

(b) Benzodiazepines

(c) Self-harm

Figure S4: Rates of psychotropic medication prescriptions and self-harm episodes – stratified by Index of Multiple Deprivation quintile (representing practices in England)

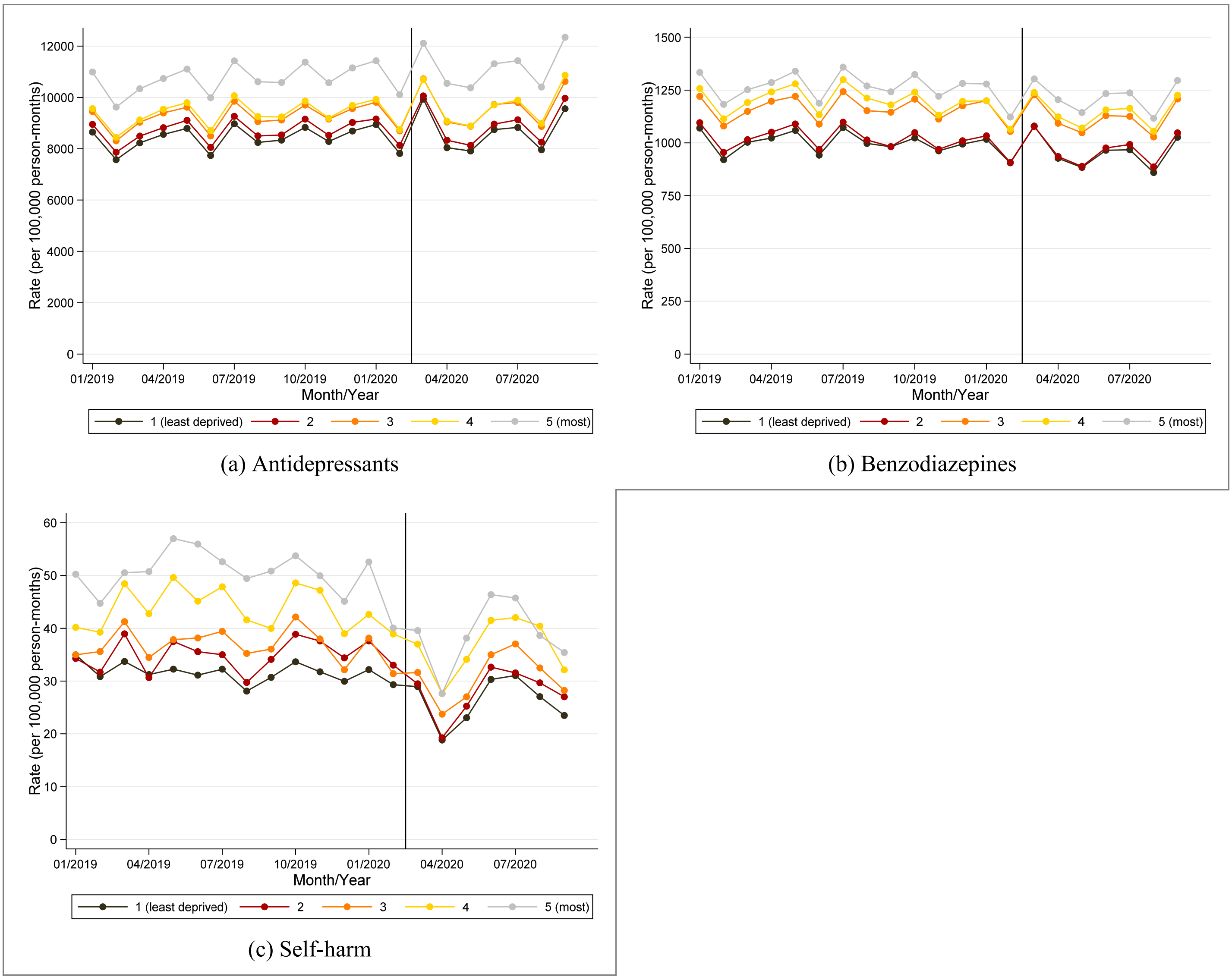

Supplementary figures from CPRD GOLD representing general practices in Northern Ireland, Scotland and Wales

Figure S5: Incident mental health diagnoses, psychotropic medication prescriptions and referrals[1] to mental health services (representing general practices in Northern Ireland, Scotland and Wales)

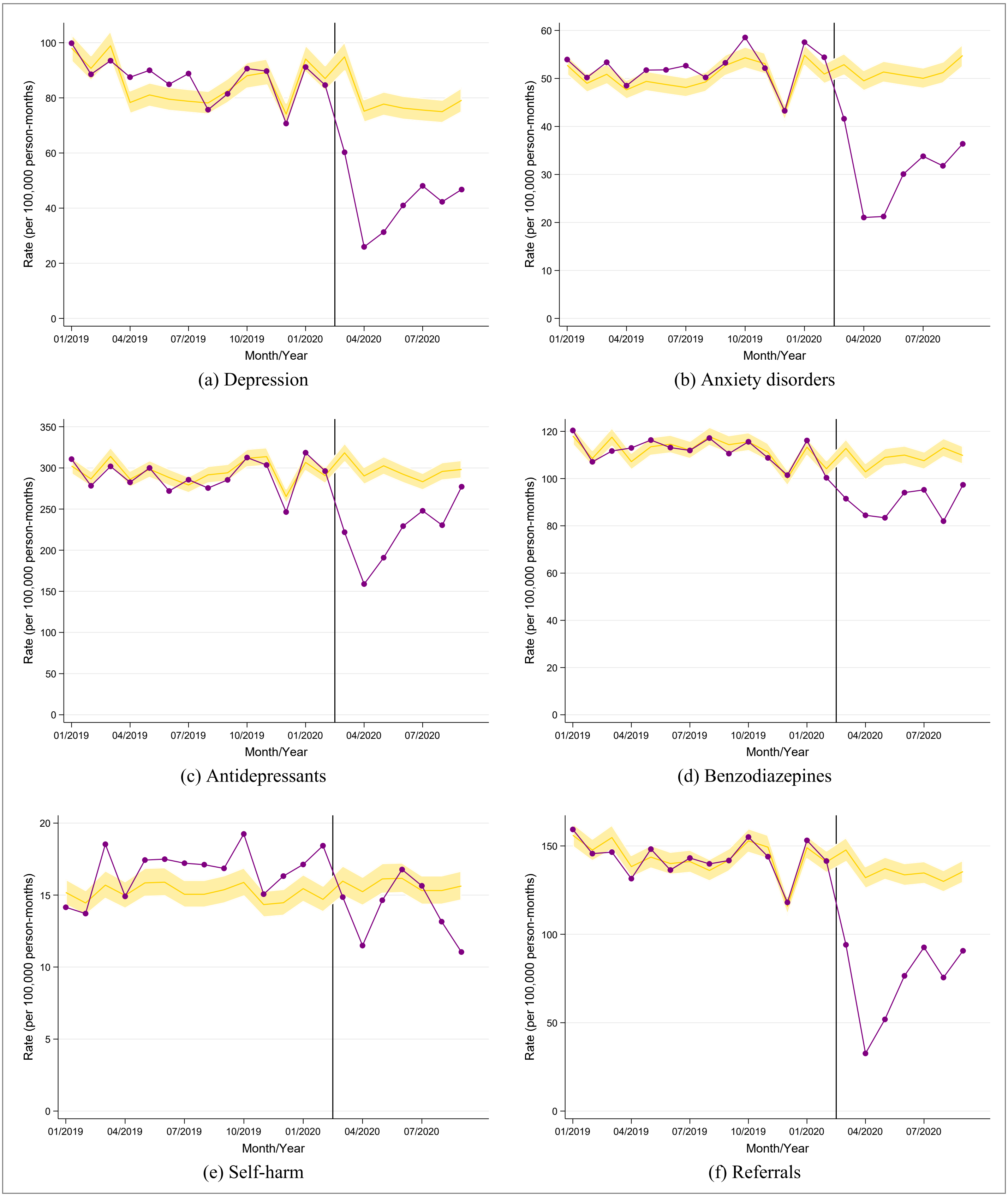

[1] The denominator for estimating rates of referral to mental health services was person-months among patients with a relevant code for depression, an anxiety disorder, or self-harm on or before the same date as their first referral.

Observed   Predicted (with shaded area representing 95% CI)

Figure S6: Incident mental illness diagnoses, psychotropic medication prescriptions and referrals[1] to mental health services – stratified by gender (representing general practices in Northern Ireland, Scotland and Wales)

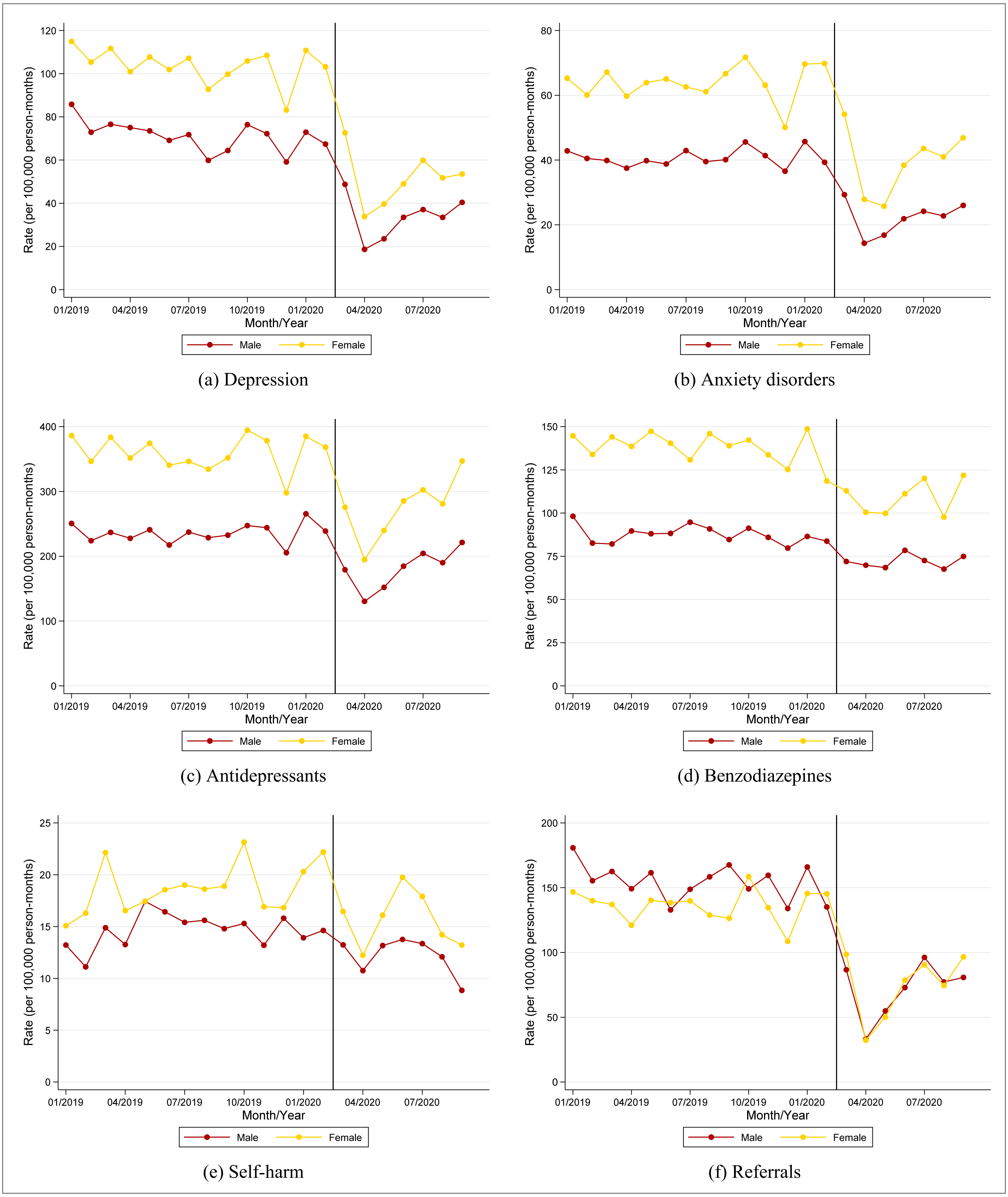

(a) Depression

(b) Anxiety disorders

(c) Antidepressants

(d) Benzodiazepines

(e) Self-harm

(f) Referrals

[1] The denominator for estimating rates of referral to mental health services was person-months among patients with a relevant code for depression, an anxiety disorder, or self-harm on or before the same date as their first referral.

Figure S7: Incident mental illness diagnoses, psychotropic medication prescriptions and referrals[1] to mental health services – stratified by age group (representing general practices in Northern Ireland, Scotland and Wales)

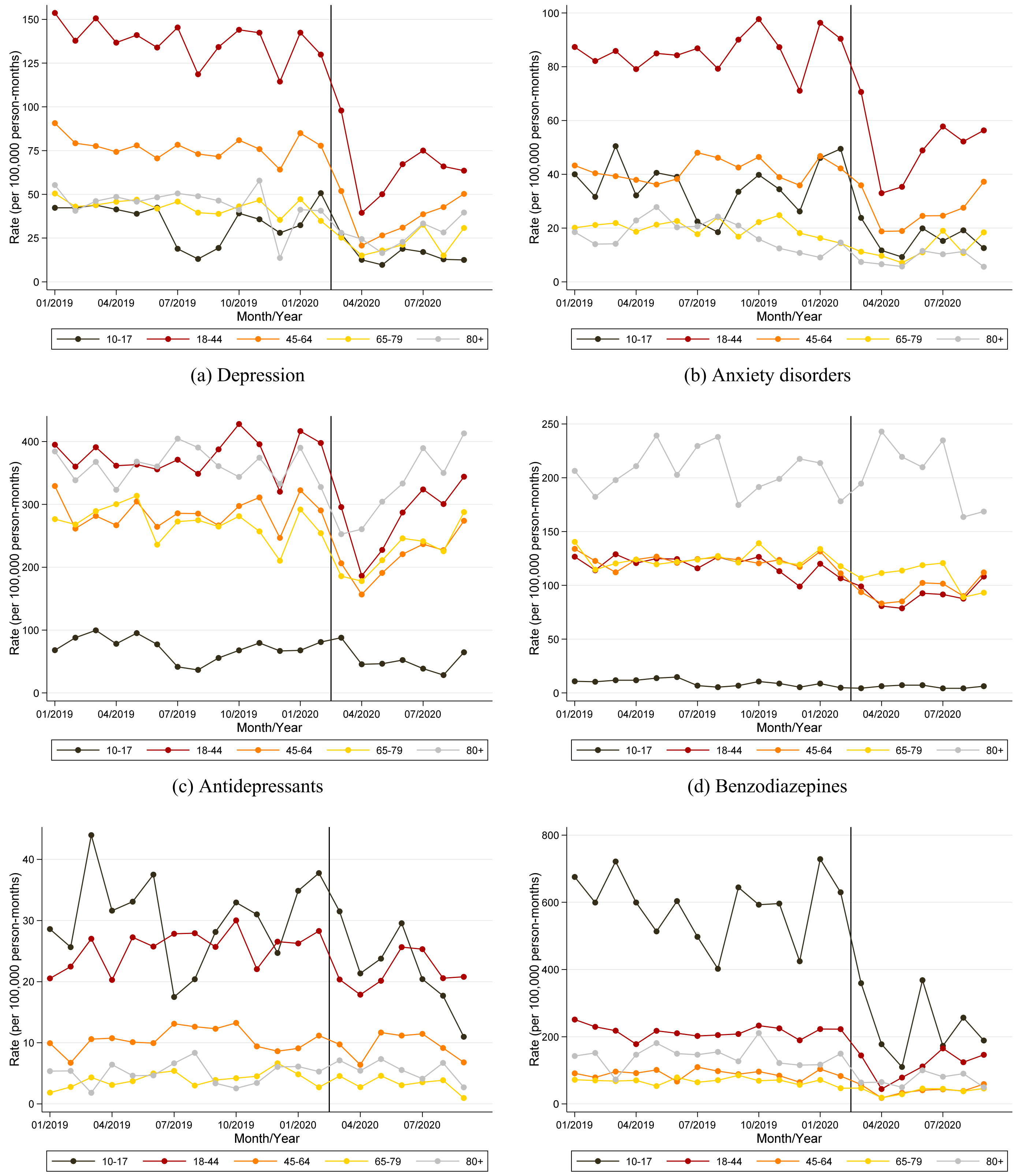

(a) Depression

(b) Anxiety disorders

(c) Antidepressants

(d) Benzodiazepines

(e) Self-harm

(f) Referrals

[1] The denominator for estimating rates of referral to mental health services was person-months among patients with a relevant code for depression, an anxiety disorder, or self-harm on or before the same date as their first referral.

Figure S8: Incident mental health diagnoses, psychotropic medication prescriptions and referrals[1] to mental health services – stratified by Index of Multiple Deprivation quintile (representing general practices in Northern Ireland, Scotland and Wales)

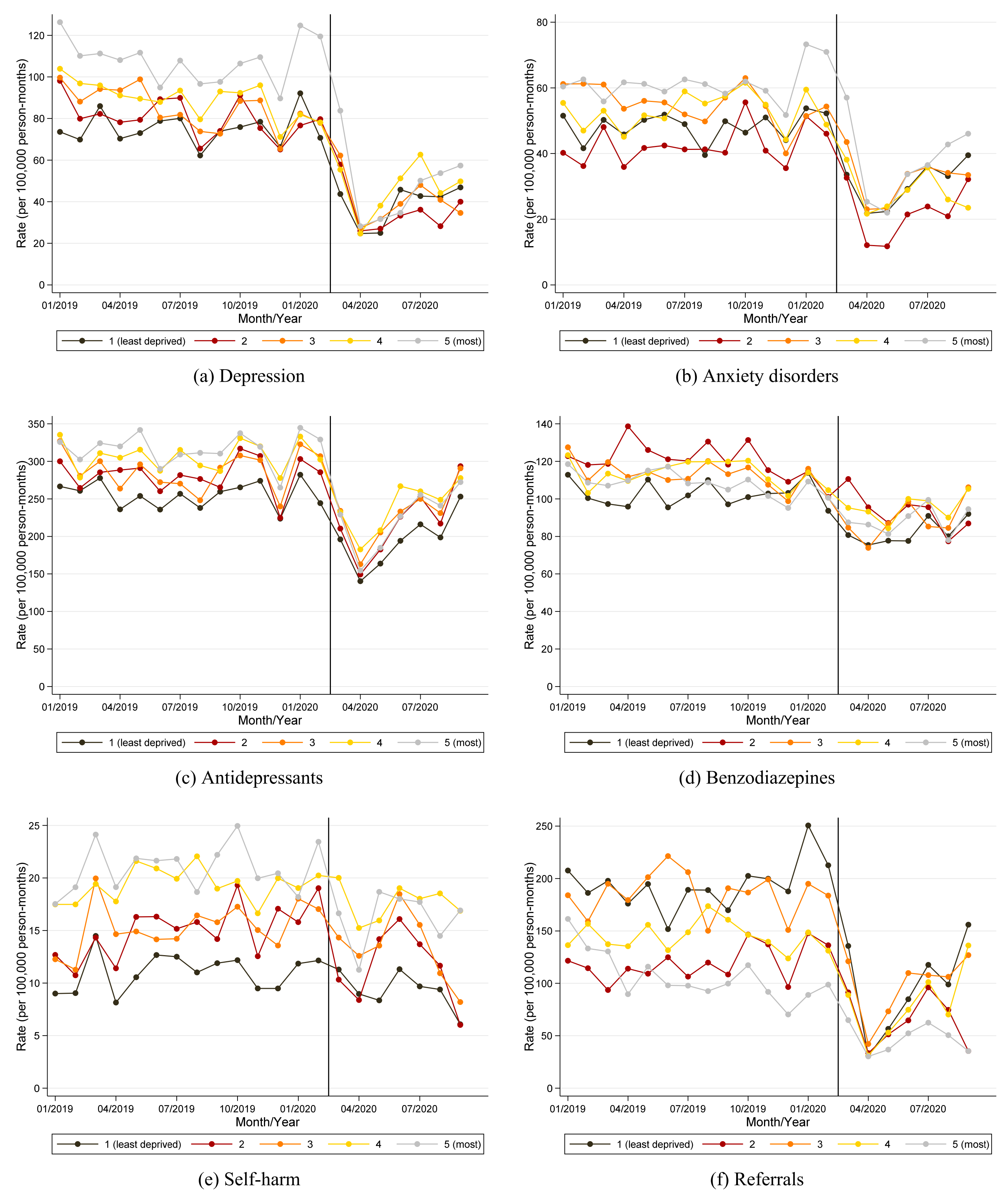

(a) Depression

(b) Anxiety disorders

(c) Antidepressants

(d) Benzodiazepines

(e) Self-harm

(f) Referrals

[1] The denominator for estimating rates of referral to mental health services was person-months among patients with a relevant code for depression, an anxiety disorder, or self-harm on or before the same date as their first referral.

Figure S9: Rates of psychotropic medication prescribing and self-harm episodes (representing general practices in Northern Ireland, Scotland and Wales)

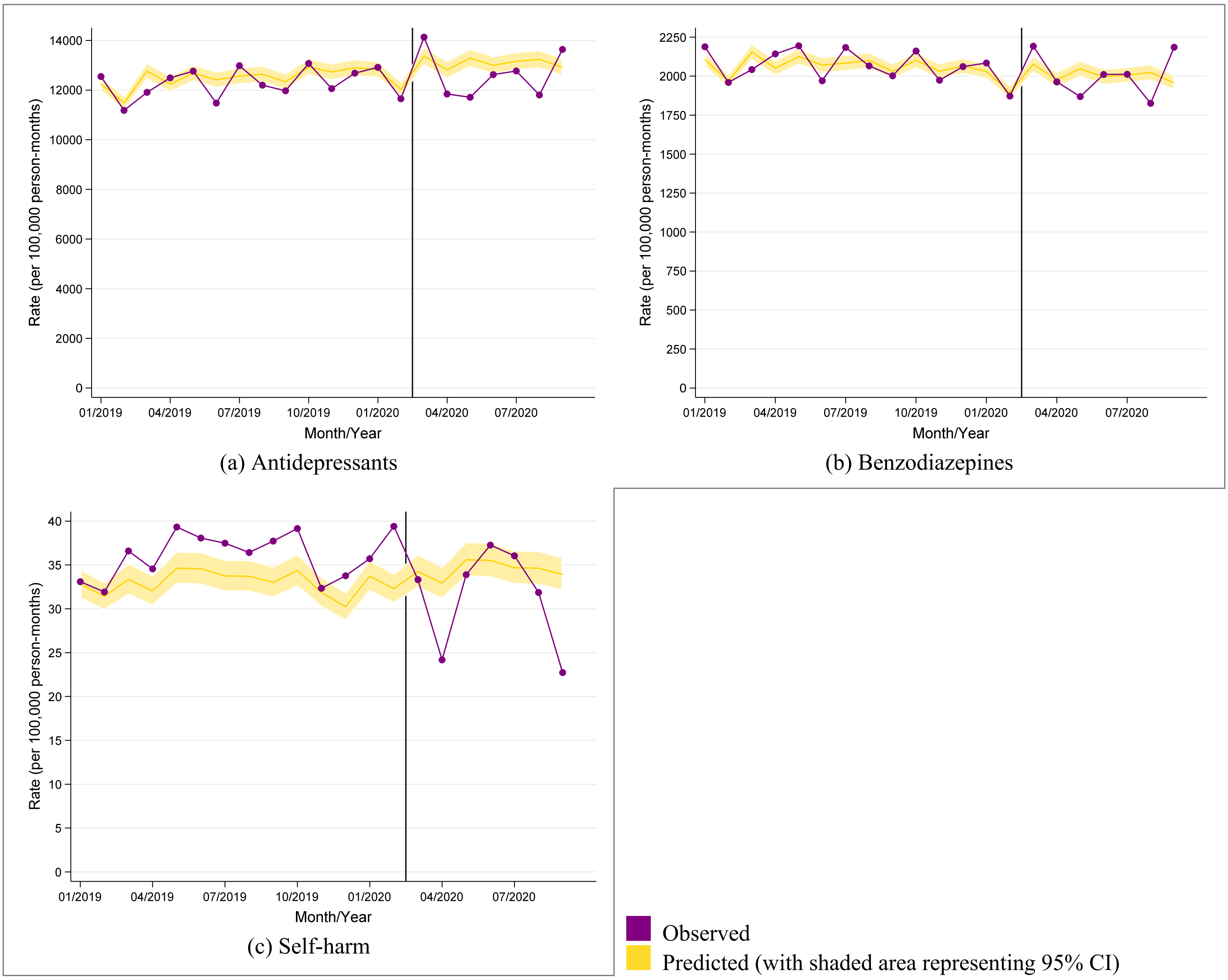

(a) Antidepressants

(b) Benzodiazepines

(c) Self-harm

Figure S10: Rates of psychotropic medication prescriptions and self-harm episodes – stratified by gender (representing general practices in Northern Ireland, Scotland and Wales)

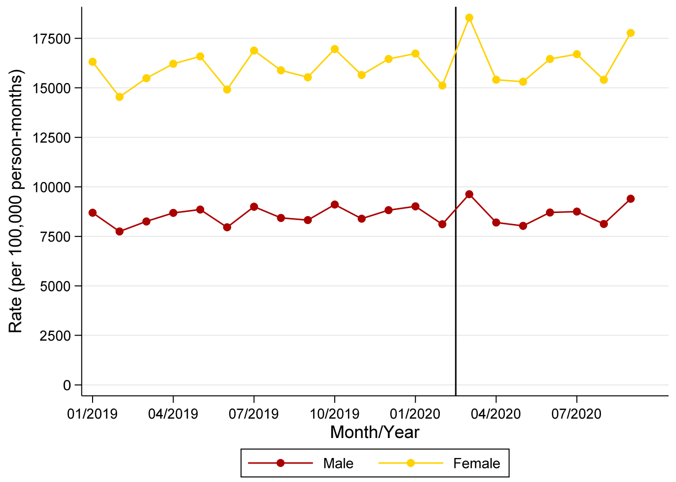

(a) Antidepressants

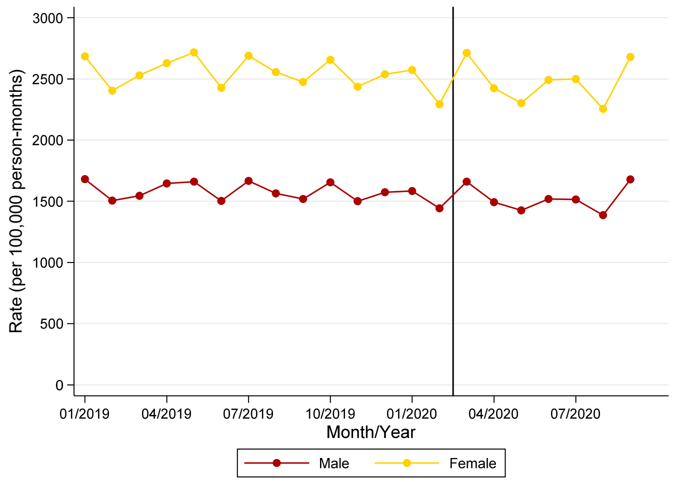

(b) Benzodiazepines

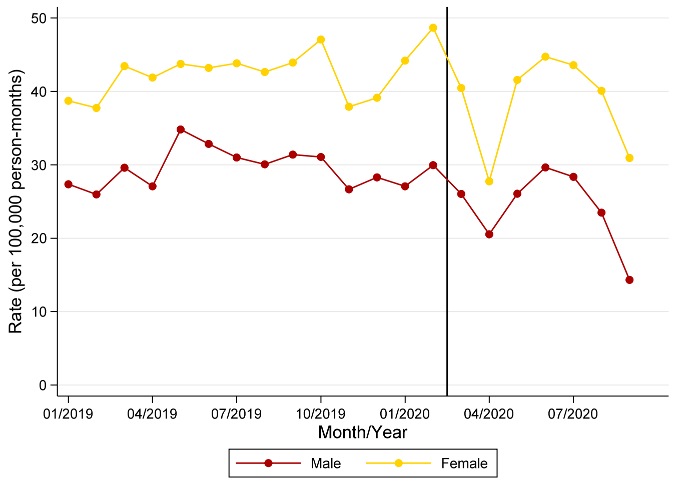

(c) Self-harm

Figure S11: Rates of psychotropic medication prescriptions and self-harm episodes – stratified by age group (representing general practices in Northern Ireland, Scotland and Wales)

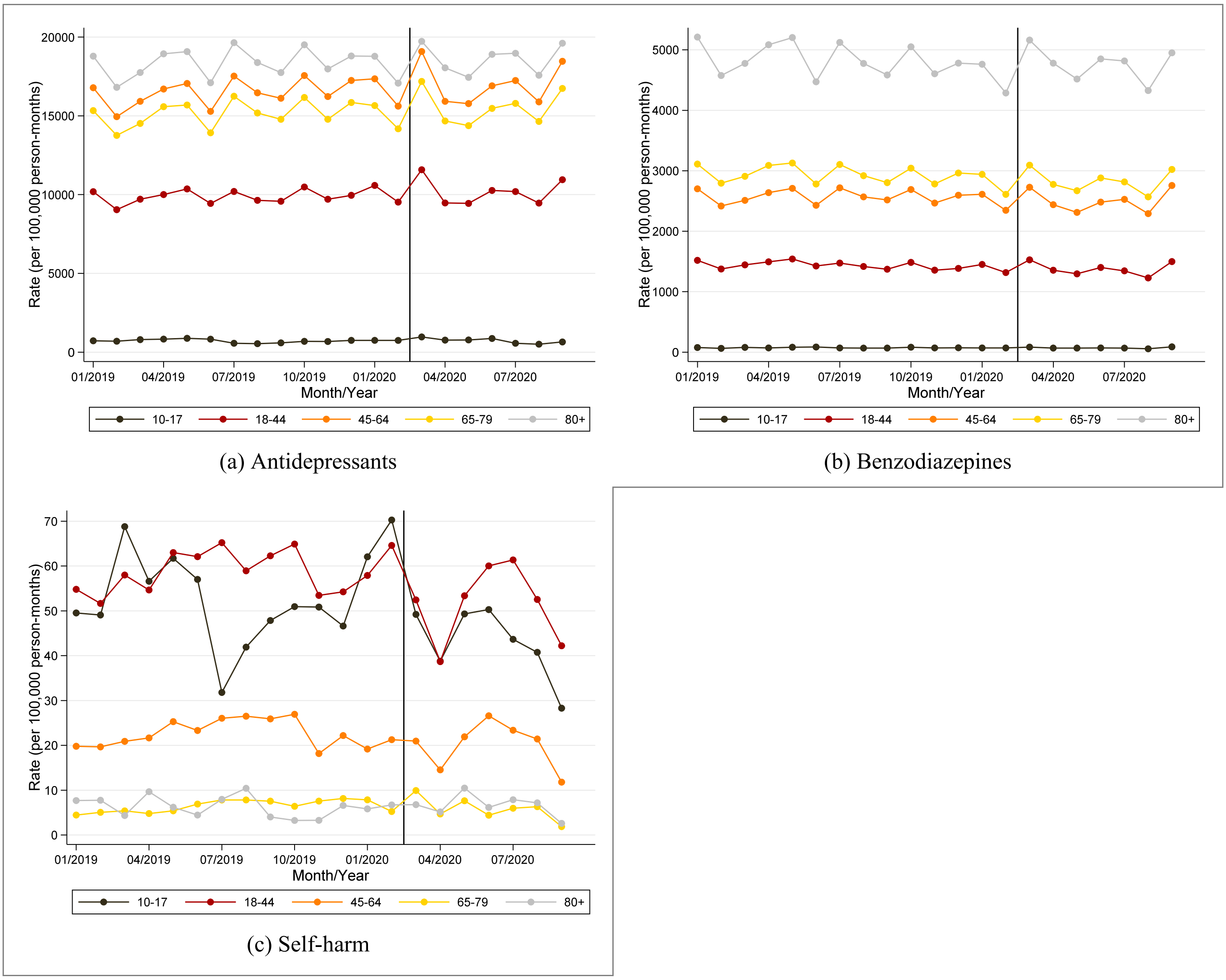

(a) Antidepressants

(b) Benzodiazepines

(c) Self-harm

Figure S12: Rates of psychotropic medication prescriptions and self-harm episodes – stratified by Index of Multiple Deprivation quintile (representing general practices in Northern Ireland, Scotland and Wales)

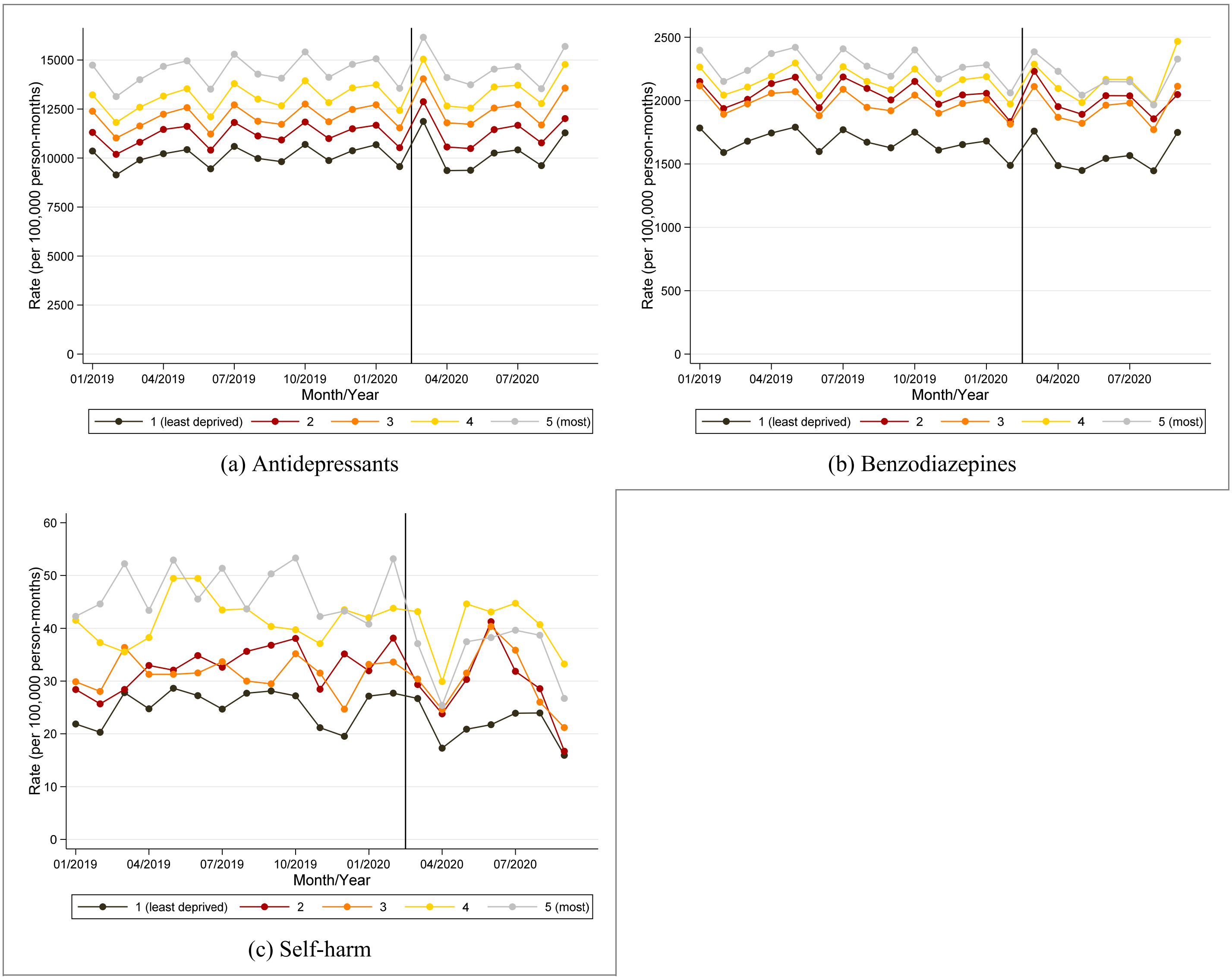

(a) Antidepressants

(b) Benzodiazepines

(c) Self-harm

Figure S13: Incident mental illness diagnoses, psychotropic medication prescriptions and self-harm episodes in England – stratified by an indicator for missing Index of Multiple Deprivation quintile

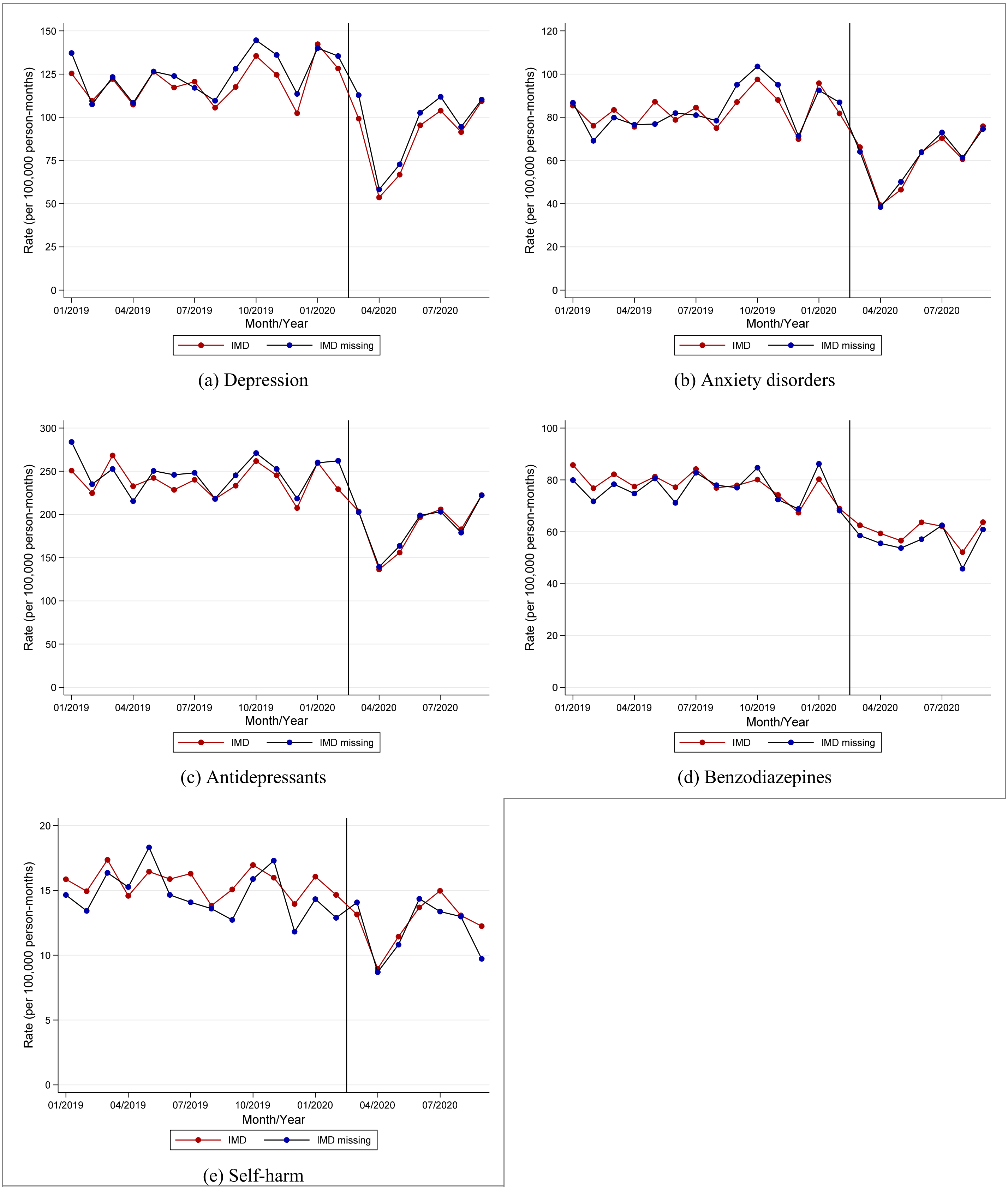

(a) Depression

(b) Anxiety disorders

(c) Antidepressants

(d) Benzodiazepines

(e) Self-harm

Table S1: Index of Multiple Deprivation quintile of the study population by nation, on 1st March 2020

	IMD quintile	*n*	%
England	1 (least deprived)	3,242,150	14.9
	2	3,785,901	17.4
	3	3,747,391	17.2
	4	4,831,553	22.1
	5 (most deprived)	4,503,115	20.6
	missing	1,712,635	7.9
Northern Ireland	1 (least deprived)	53,387	15.0
	2	57,483	16.1
	3	66,588	18.7
	4	43,981	12.4
	5 (most deprived)	116,888	32.8
	missing	17,716	5.0
Scotland	1 (least deprived)	353,932	18.2
	2	341,938	17.6
	3	364,394	18.7
	4	405,267	20.8
	5 (most deprived)	376,323	19.3
	missing	107,021	5.5
Wales	1 (least deprived)	217,881	15.9
	2	152,521	11.2
	3	315,810	23.1
	4	292,321	21.4
	5 (most deprived)	350,203	25.6
	missing	39,436	2.9

Table S2: Involvement of people with lived experience: GRIPP2 Short-form checklist

Checklist item	Description
**1: Aim**<br>Report the aim of the study	To examine trends in GP presentations for anxiety disorders, depression, self-harm episodes, antidepressant and benzodiazepine prescribing and referrals to mental health services before, during and after the peak of the Covid-19 emergency in the UK. To work with members of an existing panel of people with lived experience of self-harm and mental illness to interpret the findings.
**2: Methods**<br>Provide a clear description of the methods used for PPI in the study	Four panel members were involved in this study. Three of the members involved had prior experience of advising on research studies using electronic health records. Panel members were shown the results presented in Figure 1a with a description of what the data represented. The panel members provided their interpretation of the results by email, voice call and video call. The main points were collated and summarised by researchers before being revisited by panel members to verify accuracy.
**3: Results**<br>Outcomes—Report the results of PPI in the study, including both positive and negative outcomes	Panel members reviewed findings based on their experiences of mental illness, primary care, mental health services and the Covid-19 lockdown. Panel members then reviewed a summary of the overall interpretation of the results.
**4: Discussion**<br>Outcomes—Comment on the extent to which PPI influenced the study overall. Describe positive and negative effects	The involvement of lived experience panel members was effective in contributing to the understanding of factors influencing the rates of presentation to primary care services before, during and after the peak of the Covid-19 emergency in the UK. Specifically, panel members identified the need to compare reductions in presentations for mental illness to broader trends in primary care presentations, recognising that specific stigma exists for mental illness. Panel members and researchers held similar views about the factors contributing to reductions in help-seeking for mental illness and self-harm. Panel members offered insights into the effects government messaging to 'Stay at Home, Protect the NHS, Save Lives' had on deterring people from seeking help for mental illness.
**5: Reflections**<br>Critical perspective—Comment critically on the study, reflecting on the things that went well and those that did not, so others can learn from this experience	The panel members were able to adapt to working remotely due to the closure of workplaces following the Covid-19 pandemic. Three out of the four panel members involved had recently participated in a face-to-face workshop on mental health research involving electronic health records which may have aided their interpretation of the findings.<br><br>Panel members worked flexibly, providing written feedback and discussing their interpretation via telephone and video calls. However, the necessity for panel members and researchers to work remotely limited the depth of discussion as it was not possible to meet and discuss findings as a group.

Table S3: Demographic characteristics for patients in the study population on 1st March 2020

	**Practices in England (CPRD Aurum)**		**Practices in NI, Scotland and Wales (CPRD GOLD)**	
	n	%	*n*	%
All patients	11,432,852		2,179,560	
Gender:				
Male	5,703,748	49.9	1,078,343	49.5
Female	5,729,104	50.1	1,101,217	50.5
Age-group:				
10-17	1,161,427	10.2	214,965	9.9
18-44	4,922,913	43.1	831,017	38.1
45-64	3,235,388	28.3	667,820	30.6
65-79	1,535,563	13.4	344,909	15.8
80+	577,561	5.1	120,849	5.5
IMD quintile:				
1 (least deprived)	1,796,787	15.7	368,015	16.9
2	1,994,589	17.5	340,805	15.6
3	1,975,595	17.3	437,255	20.1
4	2,440,501	21.4	452,031	20.7
5 (most deprived)	2,295,497	20.1	477,559	21.9
Missing	929,883	8.1	103,895	4.8

Of the 21,822,745 patients from Aurum who contributed to the study population:

- 9,876,049 exited the study prior to 1st March 2020,
- 513,844 entered after 1st March 2020.

Of the 3,673,090 patients from GOLD who contributed to the study population:

- 1,409,279 exited the study prior to 1st March 2020,
- 84,251 entered after 1st March 2020.

Table S4a: Person-time and total numbers of diagnoses/events/prescriptions analysed: practices in England (CPRD Aurum)

		1st January 2010 to 29th February 2020 (Data used to model trends in expected rates)			1st March to 10th September 2020 (Data used to compare observed and expected rates)		
**Analysis type**		**Person-time (months)**	**Number of diagnoses/events**	**Number of prescriptions**	**Person-time (months)**	**Number of diagnoses/events**	**Number of prescriptions**
**Incidence**							
	Depression	1,109,047,022	1,256,462	-	60,861,067	53,443	-
	Anxiety disorders	1,159,309,593	740,709	-	64,204,857	38,189	-
	Antidepressants	964,879,657	-	2,506,295	50,954,458	-	93,845
	Benzodiazepines	1,107,265,943	-	1,223,602	61,823,764	-	36,762
	Self-harm	1,199,716,363	158,414	-	68,133,800	8512	-
**Event rates**							
	Antidepressants	1,316,677,161	-	104,849,896	74,666,902	-	7,237,716
	Benzodiazepines	1,316,677,161	-	17,750,028	74,666,902	-	818,541
	Self-harm	1,316,677,161	426,084	-	74,666,902	24,109	-

Table S4b: Person-time and total numbers of diagnoses/events/prescriptions analysed: practices in Northern Ireland, Scotland and Wales (CPRD GOLD)

		1st January 2010 to 29th February 2020 (Data used to model trends in expected rates)			1st March to 10th September 2020 (Data used to compare observed and expected rates)		
**Analysis type**		**Person-time (months)**	**Number of diagnoses/events**	**Number of prescriptions**	**Person-time (months)**	**Number of diagnoses/events**	**Number of prescriptions**
**Incidence**							
	Depression	209,795,544	215,126	-	11,017,751	4601	-
	Anxiety disorders	229,777,731	97,421	-	12,083,215	3655	-
	Antidepressants	170,085,830	-	470,434	8,353,884	-	18,081
	Benzodiazepines	199,763,747	-	271,121	10,508,919	-	9342
	Self-harm	238,713,527	33,672	-	12,673,896	1808	-
**Event rates**							
	Antidepressants	256,964,426	-	26,474,692	13,733,960	-	1,721,824
	Benzodiazepines	256,964,426	-	6,306,848	13,733,960	-	273,154
	Self-harm	256,964,426	75,535	-	13,733,960	4433	-

S5: Supplementary information on the CPRD data source

Although patterns observed for Northern Ireland, Scotland and Wales were similar to those for England, the patient management software systems and the coding classification systems that contribute to the two CPRD databases (Aurum and GOLD) are different. Therefore, variation in the identification of mental illness and self-harm between the two databases in our study is possible. Some of the reduction in primary care-recorded mental illness and self-harm may have been a result of inaccuracies in coding due to the rapid changes and adaptations that GPs had to make, including moving to using remote consultation methods, during the early stages of our study period. We did not examine depression or anxiety disorder event rates as GPs typically code a longer-term condition once. Therefore, patients may subsequently visit with symptoms of depression or anxiety, but without additional diagnostic coding. Antidepressant and benzodiazepine medications have indications beyond treating mental illness so findings relating to prescribing rates should be considered in light of this. While 98% of the population is registered at an NHS GP surgery, certain patients are not represented in our study, including prisoners, private patients, those in some residential homes and some people with no fixed address. [1 2] Due to relatively small number of primary care-recorded self-harm compared to depression and anxiety disorders, and due to the potential delay in hospital-presenting self-harm episodes being added to patients' primary care records, it is possible that not all primary care-recorded self-harm would have been captured in the latter two months of our study period (August and September 2020).

1. Herrett E, Gallagher AM, Bhaskaran K, et al. Data Resource Profile: Clinical Practice Research Datalink (CPRD). *International Journal of Epidemiology* 2015;44(3):827-36. doi: 10.1093/ije/dyv098
2. Wolf A, Dedman D, Campbell J, et al. Data resource profile: Clinical Practice Research Datalink (CPRD) Aurum. *International journal of epidemiology* 2019 doi: 10.1093/ije/dyz034

Table S6: The RECORD statement – checklist of items, extended from the STROBE statement, that should be reported in observational studies using routinely collected health data.

	Item No.	STROBE items	Location in manuscript where items are reported	RECORD items	Location in manuscript where items are reported
**Title and abstract**					
	1	(a) Indicate the study's design with a commonly used term in the title or the abstract (b) Provide in the abstract an informative and balanced summary of what was done and what was found		RECORD 1.1: The type of data used should be specified in the title or abstract. When possible, the name of the databases used should be included.<br><br>RECORD 1.2: If applicable, the geographic region and timeframe within which the study took place should be reported in the title or abstract.<br><br>RECORD 1.3: If linkage between databases was conducted for the study, this should be clearly stated in the title or abstract.	Title, page 1; Abstract: 'Methods', page 1.<br><br>Abstract: 'Methods', page 1.<br><br>N/A
**Introduction**					
Background rationale	2	Explain the scientific background and rationale for the investigation being reported			Introduction: paragraphs 1-3, pages 1-2.
Objectives	3	State specific objectives, including any prespecified hypotheses			Introduction: paragraph 3, pages 1-2.
**Methods**					
Study Design	4	Present key elements of study design early in the paper			Methods: 'Study design and data sources', page 3.
Setting	5	Describe the setting, locations, and relevant dates, including periods of recruitment, exposure, follow-up, and data collection			Methods: 'Study design, data sources and participants' and

					'Outcomes, page 3.
Participants	6	*(a) Cohort study* - Give the eligibility criteria, and the sources and methods of selection of participants. Describe methods of follow-up<br>*Case-control study* - Give the eligibility criteria, and the sources and methods of case ascertainment and control selection. Give the rationale for the choice of cases and controls<br>*Cross-sectional study* - Give the eligibility criteria, and the sources and methods of selection of participants<br><br>*(b) Cohort study* - For matched studies, give matching criteria and number of exposed and unexposed<br>*Case-control study* - For matched studies, give matching criteria and the number of controls per case		RECORD 6.1: The methods of study population selection (such as codes or algorithms used to identify subjects) should be listed in detail. If this is not possible, an explanation should be provided.<br><br>RECORD 6.2: Any validation studies of the codes or algorithms used to select the population should be referenced. If validation was conducted for this study and not published elsewhere, detailed methods and results should be provided.<br><br>RECORD 6.3: If the study involved linkage of databases, consider use of a flow diagram or other graphical display to demonstrate the data linkage process, including the number of individuals with linked data at each stage.	Methods: 'Outcomes', page 3.
Variables	7	Clearly define all outcomes, exposures, predictors, potential confounders, and effect modifiers. Give diagnostic criteria, if applicable.		RECORD 7.1: A complete list of codes and algorithms used to classify exposures, outcomes, confounders, and effect modifiers should be provided. If these cannot be reported, an explanation should be provided.	'Outcomes', page 3.
Data sources/ measuremen t	8	For each variable of interest, give sources of data and details of methods of assessment (measurement). Describe comparability of assessment methods if there is more than one group			Methods: 'Outcomes', page 3.
Bias	9	Describe any efforts to address potential sources of bias			Methods: 'Data analyses', pages 3-4.

Study size	10	Explain how the study size was arrived at			Methods: 'Outcomes' and 'Study design, data sources and participants', page 3.
Quantitative variables	11	Explain how quantitative variables were handled in the analyses. If applicable, describe which groupings were chosen, and why			Methods: 'Study design, data sources and participants', page 3.
Statistical methods	12	(a) Describe all statistical methods, including those used to control for confounding<br>(b) Describe any methods used to examine subgroups and interactions<br>(c) Explain how missing data were addressed<br>(d) *Cohort study* - If applicable, explain how loss to follow-up was addressed<br>*Case-control study* - If applicable, explain how matching of cases and controls was addressed<br>*Cross-sectional study* - If applicable, describe analytical methods taking account of sampling strategy<br>(e) Describe any sensitivity analyses			Methods: 'Data analyses', pages 3-4.
Data access and cleaning methods		..		RECORD 12.1: Authors should describe the extent to which the investigators had access to the database population used to create the study population.<br><br>RECORD 12.2: Authors should provide information on the data cleaning methods used in the study.	Methods: 'Role of funding source', page 4.

Linkage		..		RECORD 12.3: State whether the study included person-level, institutional-level, or other data linkage across two or more databases. The methods of linkage and methods of linkage quality evaluation should be provided.	Methods: 'Study design, data sources and participants' and 'Data analyses', pages 3-4
**Results**					
Participants	13	(a) Report the numbers of individuals at each stage of the study (*e.g.*, numbers potentially eligible, examined for eligibility, confirmed eligible, included in the study, completing follow-up, and analysed)<br>(b) Give reasons for non-participation at each stage.<br>(c) Consider use of a flow diagram		RECORD 13.1: Describe in detail the selection of the persons included in the study (*i.e.*, study population selection) including filtering based on data quality, data availability and linkage. The selection of included persons can be described in the text and/or by means of the study flow diagram.	Methods; 'Data analyses' and 'Results', page 4.
Descriptive data	14	(a) Give characteristics of study participants (*e.g.*, demographic, clinical, social) and information on exposures and potential confounders<br>(b) Indicate the number of participants with missing data for each variable of interest<br>(c) *Cohort study* - summarise follow-up time (*e.g.*, average and total amount)			Appendix p17-18.<br><br>Methods, 'Data analyses', page 3-4 and Appendix p15.
Outcome data	15	*Cohort study* - Report numbers of outcome events or summary measures over time<br>*Case-control study* - Report numbers in each exposure category, or summary measures of exposure<br>*Cross-sectional study* - Report numbers of outcome events or summary measures			Results, pages 4-5 and Tables 1a and 1b.
Main results	16	(a) Give unadjusted estimates and, if			Results, pages 4-5.

		applicable, confounder-adjusted estimates and their precision (e.g., 95% confidence interval). Make clear which confounders were adjusted for and why they were included<br>(b) Report category boundaries when continuous variables were categorized<br>(c) If relevant, consider translating estimates of relative risk into absolute risk for a meaningful time period			
Other analyses	17	Report other analyses done—e.g., analyses of subgroups and interactions, and sensitivity analyses			Results, page 5 and Appendix.
**Discussion**					
Key results	18	Summarise key results with reference to study objectives			Discussion, paragraph 1, page 6.
Limitations	19	Discuss limitations of the study, taking into account sources of potential bias or imprecision. Discuss both direction and magnitude of any potential bias		RECORD 19.1: Discuss the implications of using data that were not created or collected to answer the specific research question(s). Include discussion of misclassification bias, unmeasured confounding, missing data, and changing eligibility over time, as they pertain to the study being reported.	Discussion, page 10.
Interpretation	20	Give a cautious overall interpretation of results considering objectives, limitations, multiplicity of analyses, results from similar studies, and other relevant evidence			Discussion: pages 6-11.
Generalisability	21	Discuss the generalisability (external validity) of the study results			Discussion, page 10.
**Other Information**					
Funding	22	Give the source of funding and the role of the funders for the			Methods, 'Role of funding

		present study and, if applicable, for the original study on which the present article is based			source', page 4 and 'Acknowledgements', page 11.
Accessibility of protocol, raw data, and programming code		..		RECORD 22.1: Authors should provide information on how to access any supplemental information such as the study protocol, raw data, or programming code.	Data sharing, page 11.

*Reference: Benchimol EI, Smeeth L, Guttmann A, Harron K, Moher D, Petersen I, Sørensen HT, von Elm E, Langan SM, the RECORD Working Committee. The REporting of studies Conducted using Observational Routinely-collected health Data (RECORD) Statement. *PLoS Medicine* 2015; in press.

*Checklist is protected under Creative Commons Attribution (CC BY) license.
